# Household representatives’ understanding of dengue solutions and larval indices surveillance systems in southern Thailand: A mixed methods study

**DOI:** 10.1371/journal.pone.0348142

**Published:** 2026-10-08

**Authors:** Charuai Suwanbamrung, Kusubeeyah Babathee, Sikarin Aiedchuthong, Juthaphon Detaran, Farusdeen Madsen, Lafi Munira, Dereje Haile, Aseb Arba Kinfe

**Affiliations:** 1 Doctor of Philosophy Program in Public Health Research, School of Public Health, Walailak University, Nakhon Si Thammarat, Thailand; 2 Excellent Center for Public Health Research (EC forPHR), Walailak University, Nakhon Si Thammarat, Thailand; 3 College of Health Sciences and Medicine, Wolaita Sodo University, Wolaita Sodo, Ethiopia; Chiang Mai University Faculty of Agriculture, THAILAND

## Abstract

**Background:**

Dengue fever remains a major public health concern in tropical regions, particularly in Southeast Asia. Effective prevention depends on the community’s understanding of both dengue control measures and vector surveillance systems. This study assessed and explored household representatives’ understanding of dengue solutions (care and preventive measures) and the *Aedes* larval index surveillance system in southern Thailand.

**Methods:**

A community-based convergent mixed-methods study was conducted among 824 household representatives selected through stratified random sampling. Quantitative data were collected using a structured questionnaire and analyzed using logistic regression to identify factors associated with understanding. Qualitative data were collected through in-depth interviews with 13 purposively selected participants and analyzed using thematic analysis. Findings were integrated using a joint display approach.

**Results:**

Only 28.9% of participants demonstrated a high understanding of the dengue solution, and 1.2% had a high understanding of the larval indices surveillance system. Training attendance (AOR 2.01, 95% CI: 1.45–2.78), fewer water containers (AOR 2.06, 95% CI: 1.20–3.54), and higher larvae-positive containers (AOR 0.64, 95% CI: 0.43–0.97) were significantly associated with lower understanding. Qualitative findings revealed the following key themes: recognition of dengue fever, awareness of preventive practices, risky season, and the importance of training. Training was identified as a key factor in improving understanding and practices.

**Conclusion:**

Household heads’ understanding of dengue prevention is limited, particularly regarding larval surveillance systems. Household representatives were familiar with dengue symptoms; they lacked technical knowledge of surveillance systems. Strengthening community-based training and improving the communication of technical concepts are essential to enhance dengue prevention and control efforts.

## 1 Introduction

Dengue is a mosquito-borne viral disease caused by antigenically related dengue virus serotypes (DENV 1–4) and remains one of the most important vector-borne diseases worldwide. Dengue fever is endemic in more than 100 countries, particularly in tropical and subtropical regions, with Southeast Asia representing the global epicenter of dengue fever [1–3]. Over the past two decades, the global burden of dengue has increased substantially, with reported cases rising from half a million in 2000 to 5.2 million in 2019 [4,5]. Following a temporary decline during the COVID-19 pandemic, dengue resurged dramatically, with more than 6 million cases and over 6,000 deaths reported worldwide in 2023, representing the largest epidemic on history [4,6].

In Asia, there were more than 1.6 million cases and 3,637 deaths in 2023 [7]. The majority of the Asian population, particularly in Southeast Asia, is estimated to be living in areas where dengue transmission susceptibility is high [5,8]. Thailand is one of the 30 countries with the highest dengue fever endemicity, where transmission is influenced by serotype shifts, climatic conditions, and vector ecology. Although the dengue incidence declined during the COVID-19 period, the number of reported cases increased again in 2022, with Southern Thailand continuing to experience a considerable burden of disease [3,9,10]. According to the WHO South-East Asia Regional Office Epidemiological Bulletin, Thailand reported approximately 46,000–47,000 dengue cases in 2022. In 2023, dengue cases increased markedly to 150,808, with 165 deaths reported by December. This was the highest dengue burden recorded in Thailand since 2018, highlighting the resurgence of dengue transmission [11].

Southern Thailand remains a dengue-endemic region characterized by recurrent seasonal outbreaks and persistent *Aedes aegypti* breeding sites within the community [8,12]. Previous surveillance and operational research conducted in the region, including the development of the “Laskan Model” and community-based larval index surveillance systems, have demonstrated that dengue transmission is closely associated with household water storage practices and environmental management [12]. Despite control efforts, the persistence of household breeding sites and recurrent outbreaks highlights the importance of community participation in dengue prevention and larval surveillance. Understanding household representatives’ knowledge of dengue prevention measures and larval surveillance systems is therefore essential for strengthening community-based dengue control programs in endemic settings such as southern Thailand [12,13].

Dengue fever can be prevented through vector control, source reduction, personal protection, and community engagement in surveillance and monitoring. Nevertheless, the effectiveness of prevention depends on the community’s and other stakeholders’ understanding and action of the recommended dengue fever solutions and the larval indices surveillance system [10,13–17]. Previous studies conducted in Thailand have reported moderate to good understanding of dengue prevention among community members, although preventive practices often remain suboptimal, while poor understanding has been reported among high school students, particularly regarding larval surveillance concepts [18–20].

According to Bloom’s revised taxonomy, understanding is a cognitive process that enables individuals to interpret, apply, and translate knowledge into practice [21]. In the context of dengue control, understanding of disease transmission, symptoms and management, vector ecology, and surveillance strategies is essential for effective household-level prevention. Previous studies have shown that understanding is influenced by educational attainment, access to health information, socioeconomic status, prior dengue experience, participation in community programs, and dengue-related training [13,15,16,22]

Sichon District represents an environment where favorable climatic conditions for *Aedes* mosquito breeding, recurrent dengue outbreaks, and an active community network intersect. Household representatives’ understanding of dengue solutions and the *Aedes* larval index surveillance system is crucial, as it enables them to take responsibility for early treatment, manage their environment effectively, and educate family members and neighbors on controlling the breeding sites of *Aedes* mosquitoes. However, limited research has investigated household representatives’ understanding of dengue solutions or care and preventive measures, and larval surveillance systems among household representatives, who are the primary role players in environmental management indoors and outdoors.

Therefore, this study aimed to fill the gap through achieving the following objectives: 1) To describe the household representatives’ understanding of dengue solutions or care and preventive measures and the *Aedes* mosquito larvae index surveillance system; 2) To identify factors associated with household representatives’ understanding of dengue solutions and the *Aedes* mosquito larvae index surveillance system; 3) To explore the household representatives’ understanding of dengue solutions and the *Aedes* mosquito larvae index surveillance system.

## 2 Methods and materials

### 2.1 Research design

This study employed a community-based, convergent parallel mixed-methods design to assess the understanding of dengue solutions and the *Aedes* Mosquito Larvae Surveillance System among household representatives in Sichon District, Nakhon Si Thammarat Province. Data were collected concurrently for a better explanation of household representatives’ understanding of both dengue fever solutions and the *Aedes* Mosquito Larvae Index surveillance system, the associated factors, their experience with dengue solutions, and the *Aedes* mosquito larvae index surveillance system from November 15, 2023, to January 30, 2024.

### 2.2 Setting

Sichon District is one of the northernmost districts of Nakhon Si Thammarat and is located along the eastern coast of southern Thailand, bordering the Gulf of Thailand. The district covers both coastal lowlands and hilly inland areas, with a tropical monsoon climate that provides favorable conditions for *Aedes* mosquito breeding throughout the year. Sichon District has a predominantly rural population engaged in agriculture, fishing, and small-scale commerce, with scattered urban centers serving as local hubs for education and healthcare. The district’s public health infrastructure includes subdistrict health-promoting hospitals and village health volunteers, who play a central role in dengue surveillance and prevention activities. Owing to its climatic and ecological conditions, the Sichon district has experienced recurrent dengue outbreaks, making it a suitable and representative setting for studying community understanding and participation in the *Aedes* mosquito larvae index surveillance system. The study area comprises 9 subdistricts with 27,767 households.

### 2.3 Population and eligibility

The population was household representatives aged 20 years and older residing in Sichon District, Nakhon Si Thammarat Province.

#### 2.3.1 Inclusion and exclusion criteria.

The inclusion criteria for participants were as follows: Household representatives aged 20 years or older, residing at a registered address in Sichon District, Nakhon Si Thammarat Province, and who consented to participate and provide information participated in the quantitative study, whereas household representatives who had experience with dengue fever and the larval index surveillance system and were willing to participate in the in-depth interview were involved in the qualitative study.

### 2.4 Sample size determination

The quantitative sample size was determined as part of the planning of the broader dengue prevention implementation project that followed the baseline mixed methods assessment. It was calculated using the GPower 3.1 program, with an effect size of 0.15 (small effect), a significance level of 0.05, and a power of 0.95. The degrees of freedom were set at 2, and the effect size was determined based on the research findings, which reported that 33.9% of participants had a good understanding before training [23], This was used to estimate that exposure to information would improve understanding by 7%. Using these parameters, the GPower analysis produced a required sample size of 687 participants. To accommodate an expected 20% nonresponse rate and reduce potential errors, the final sample size was increased to 824 participants. A total of 824 household representatives were therefore included in the quantitative baseline assessment. The sample size of the qualitative study was 13 household representatives, and the interviews were stopped because of information saturation.

### 2.5 Sampling method

The stratified random sampling technique was employed to ensure that the participants selected were representative of all districts within the study area. This method allowed for the inclusion of diverse household characteristics across the district while minimizing sampling bias. The total households in the Sichon district were stratified into households under subdistrict health-promoting hospitals. The total number of participants was proportionally allocated to each subdistrict health-promoting hospital on the basis of the number of households residing in that subdistrict health-promoting hospital, as shown in the Supplementary file. Within each stratum, households in randomly selected villages were involved in the study to maintain random participant inclusion.

A purposive sampling technique was used to select participants for in-depth interviews to obtain information directly relevant to the research objectives. The key informants were household representatives who had experience with dengue fever and the larval index surveillance system. Participation was voluntary, and the number of informants was determined based on data saturation, meaning that data collection continued until participant 13, when no new information or themes emerged.

### 2.6 Data collection tools

The researchers developed a questionnaire based on a review of the relevant literature and reviewed by experts, ensuring that each question aligned with the research objectives and variables of interest. The quantitative data collection questionnaire comprises four sections as follows:

**Personal Information**: includes gender, age, religion, marital status, education level, occupation, history of dengue fever, dengue history among family members, and participation in dengue-related training within the past 12 months.
**Environmental conditions:** comprises housing characteristics, water usage systems, waste management practices, and larval index data.
**Understanding of Dengue Solutions (UDS):** The questionnaire consisted of 10 multiple-choice items focusing on dengue, causes, symptoms and signs, treatment, and prevention with five answer options for each question, which were later converted to correct (1) and incorrect responses (0). The understanding levels were scored as low when the respondent’s score was less than 80% or high if the respondent’s score was equal to or greater than 80% [13,20].
**Understanding of the Dengue Larval Index Surveillance System (UDLSS):** This section consisted of 10 multiple-choice items comprising the Container Index (CI), the meaning of larval indices, mosquito breeding site elimination, individuals responsible for larval surveys, and methods for larval inspection, with five answer options for each question, which were later converted to correct (1) and incorrect responses (0). This tool was developed from the local larval surveillance system operational procedure and validated by earlier studies. An understanding level of the *Aedes* mosquito larval index surveillance system was classified as low if the respondent scored less than 80% and high if the household representative answered 80% or more of the questions correctly [13,20].

The researchers developed an in-depth interview guide based on a comprehensive review of the relevant literature, ensuring alignment with the research objectives. The in-depth interview questionnaire included sections on basic personal details and experiences related to dengue fever, insights into environmental assessments, and practices related to dengue fever and the larval index surveillance system.

### 2.7 Definition of terms

**Understanding of Dengue Solution**: refers to the respondents’ ability to identify key concepts related to dengue fever, including its definition, causes, signs and symptoms, treatment, and prevention. This understanding was assessed using a ten-item questionnaire.
**Understanding of the Larval Index Surveillance System**: refers to the respondents’ ability to identify essential aspects of the larval index surveillance system, such as the Container Index (CI), the meaning of larval indices, mosquito breeding site elimination, individuals responsible for larval surveys, and methods of larval inspection. This understanding was measured using a ten-item questionnaire.
**Pathogen-related Factors**: refer to the causative agent of dengue fever; the dengue virus is transmitted by *Aedes* mosquitoes, which is measured through the larval index, which indicates mosquito density in a given area using the Container Index (CI). The acceptable standard value should not exceed 1%.
**Household Representative:** Refers to an individual recognized by household members as responsible for the financial management, welfare, and health care of household members. The household representative may not necessarily be the house owner or the parents, but could be any family member who assumes these responsibilities.
**Surveillance System:** refers to a systematic and continuous process of monitoring, recording, analyzing, and interpreting data related to disease occurrence and distribution, public health events, or problems. The surveillance process includes data collection, organization, analysis, and dissemination of findings to support effective planning, prevention, and control measures for public health management.

### 2.8 Quality verification of instruments

The research instrument was designed to achieve the research objectives and was checked for its consistency and comprehensive coverage of all relevant research variables in consultation with subject matter experts related to the study. The interview guide was developed based on a review of various studies to ensure its alignment with the research objective. The content validity of the questionnaire and interview guide was examined by three experts, including a nursing lecturer from Walailak University, a public health specialist from the Office of Disease Prevention and Control in Nakhon Si Thammarat Province, and a public health officer from a subdistrict health-promoting hospital.

The pilot study of the research instrument was tested with 30 household representatives in Sichon District, Nakhon Si Thammarat Province. Because UDS and UDLSS were scored dichotomously, internal consistency was assessed using the Kuder-Richardson Formula 20. KR-20 was calculated separately for both UDS and UDLSS. The UDS showed good reliability (0.855), whereas UDLSS items had moderate internal consistency (0.65). However, the researchers identified items affecting the internal consistency in UDLSS and revised them before conducting the main study. Both tools had previously undergone language adaptation and pretesting in a population comparable to the study population using the same language and administration format.

The final version of the research instrument was used for data collection in this study. The triangulation of the responses of household representatives who participated in the qualitative study was done through observation of water containers indoors and outdoors, and the environmental conditions surrounding their households. The research team members were invited to check for consistency of findings and to ensure the rigor of the study.

### 2.9 Data collection procedure

Quantitative data were collected using a Thai version of a structured questionnaire in collaboration with village health volunteer leaders (VHLs) from each subdistrict health-promoting hospital to aid in data collection from household representatives. Training sessions were conducted for voluntary health leaders during their monthly meetings at each subdistrict health-promoting hospital. VH leaders then guided the data collectors by showing the direction of randomly selected household representatives in their respective villages according to the inclusion criteria. Strict supervision was performed to manage incomplete data reports on time.

The researchers conducted an in-depth interview in Thai by scheduling sessions depending on the participants’ availability, with each session lasting approximately 45–60 minutes to minimize disruption. Field notes with an observation checklist were used to record the response of the participants. The interviews were conducted until data saturation was achieved, which occurred with the 13^th^ participant, indicating that no new insights emerged and marking the end of the interview process.

### 2.10 Ethical approval and consent

This study was conducted in accordance with the Declaration of Helsinki and approved by the Ethical Review Board of Walailak University (Project No. WU-EC-PU-1-259-66), and Approval No. WUEC-23-277-01. Informed consent was obtained from each household representative before conducting the interview.

### 2.11 Data analysis

The researcher reviewed all the completed questionnaires for completeness and organized the data systematically. The data in the spreadsheet were exported to the Statistical Package for Social Sciences software for analysis. Continuous variables were then categorized, and latent variables were computed from the collected data. Descriptive statistics were performed for both personal and environmental variables, which are presented as frequencies and percentages. The dependent variables were derived from the specific questions designed to measure each respective construct.

Binary logistic regression was used to identify factors associated with household representatives’ understanding of dengue solutions (UDS) and the larval index surveillance system (UDLSS). Initially, bivariate analyses were conducted to assess each independent variable and the outcome variables. Variables with p-values less than 0.25 in the bivariate analysis were considered candidates for multivariate analysis to avoid excluding potentially important predictors.

Multivariate logistic regression models were then constructed using the Enter approach. Adjusted odds ratios (AORs) with 95% confidence intervals (CIs) were reported to estimate the strength and direction of associations. Statistical significance was set at p < 0.05. Before model fitting, multicollinearity among independent variables was assessed using the variance inflation factor (VIF). Model fitness was evaluated using the Hosmer-Lemeshow goodness-of-fit test. The assumptions of logistic regression were checked. Because only ten (1.2%) participants met the predefined criterion for high UDLSS, there were insufficient outcome events to support reliable logistic regression modelling for this outcome. Therefore, UDLSS was analyzed descriptively, and no regression analysis was conducted to identify factors associated with high UDLSS.

Qualitative data were analyzed using a thematic analysis approach following an inductive coding process. All interviews were transcribed verbatim and reviewed multiple times to ensure familiarity with the data. Initial codes were generated from meaningful segments of the text, and similar codes were grouped into categories based on conceptual similarities. The categories were then synthesized into broader themes that reflected participants’ experience and understanding of dengue prevention and larval surveillance.

### 2.12 Integration of the quantitative and qualitative findings

Integration of quantitative and qualitative findings was conducted using a convergent parallel mixed-methods approach. After separate analysis, findings were compared and merged through a joint display technique to identify areas of convergence, complementarity, and divergence. This approach enabled the development of meta-inferences by linking statistical associations with participants’ experiences and perceptions. The qualitative findings were used to explain and contextualize the quantitative results, particularly in understanding the underlying reasons for low levels of knowledge.

## 3 Results

### 3.1 Sociodemographic and personal information of the respondents

This study involved all 824 targeted household representatives, achieving a complete response rate. Most participants were female (73.1%), with an average age of 48 ± 12.57 years, and the majority (57.6%) were in the 40–59 years age group. In terms of demographics, most respondents were Buddhists (95.6%) and were married (70.6%). Regarding education level, the largest group had primary education and lower (45.4%), followed by those with upper secondary education (30.1%). In terms of occupation, the majority were farmers (44.5%), followed by general laborers (22.8%), as summarized in Table 1.

**Table 1:** Sociodemographic characteristics of the household representatives who participated in the study (n=824).

Personal Information	Category	Number (Percentage)
Gender	Male	222 (26.9)
	Female	602 (73.1)
Age	20–39	191 (23.2)
	40–59	475 (57.6)
	60 years and over	158 (19.2)
Religion	Buddhist	788 (95.6)
	Muslim	33 (4.0)
	Christian	3 (0.4)
Marital status	Single	153 (18.6)
	Married	582 (70.6)
	Widowed/Divorced	89 (10.8)
Education level	Primary and lower	374 (45.4)
	Secondary school	248 (30.1)
	Associate Degree/ Vocational Certificate	64 (7.8)
	Bachelor’s degree and above	138 (16.7)
Occupation	Unemployed	70 (8.5)
	General laborer	188 (22.8)
	Farmer	367 (44.5)
	Self-employed	142 (17.2)
	Government or enterprise employed	57 (6.9)

### 3.2 Dengue fever-related experiences

With respect to the experience of dengue fever infection among household heads and family members in the study area, almost all participants and their family members (96.7%) had no previous experience with dengue fever. Furthermore, more than one-third of the participants had attended at least one dengue fever-related training session in the past 12 months, as shown in Table 2.

**Table 2:** Experience related to dengue fever infection and training among household representatives in Sichon District, Nakhon Si Thammarat.province.

Variables	Category	Frequency (percentage)
Experience of dengue fever infection (past 12 months)	No	797 (96.7)
	Yes	27 (3.3)
Family history of dengue fever infection (past 12 months)	No	797 (96.7)
	Yes	27 (3.3)
Attended training on dengue fever (past 12 months)	No	540 (65.5)
	Yes	284 (34.5)

### 3.3 Mosquito breeding sites inside and outside the house

The household container survey identified a total of 2,782 containers inside homes, of which 584 contained *Aedes* larvae. Outside the house, 2,907 containers were recorded, and 1,098 of these containers were positive for larvae. When categorized by type, water storage containers were the most prevalent breeding sites, totaling 1,016 indoors and 988 outdoors. The greatest number of *Aedes* larvae was found in outdoor water storage containers for household utilization, with 305 containers testing positive, as shown in Table 3.

**Table 3:** Types and number of containers potentially serving as mosquito breeding sites indoors and outdoors (n = 2   4).

Types of Containers Holding Water	Indoors		Outdoors	
	Total	Larvae Found	Total	Larvae Found
1. Water storage containers for household utilization	1016	171	988	305
2. Drinking water containers	774	85	325	96
3. Ant-proof cabinet trays	460	104	122	54
4. Plant saucers	116	39	403	133
5. Flower vases	200	68	143	59
6. Car tires	75	36	266	145
7. Water basins for lotus or ornamental plants	64	32	318	120
8. Unused containers	77	49	342	186
Total	2782	584	2907	1098

### 3.4 Larval indices in the subdistricts of Sichon District

All nine subdistricts in Sichon District were identified as being at risk of dengue fever outbreaks. Analysis of the larval indices revealed that the breteau index (BI) ranged from 88 to 332 (standard BI ≤ 50), the house index (HI) ranged from 27 to 62 (standard HI ≤ 10), and the container index (CI) ranged from 13 to 30 (standard CI ≤ 1) (in Supplementary file). Among all subdistricts, Thung Prang recorded the highest larval indices compared with the other subdistricts.

#### 3.4.1 Levels of the container index (CI).

The analysis of Container Index levels revealed that the majority, 490 (59.5%) households, had CI values within the standard threshold (standard criterion: CI ≤ 1). In contrast, 334 households (40.5%) had CI values exceeding the standard, as shown in Fig 1.

**Fig 1:**
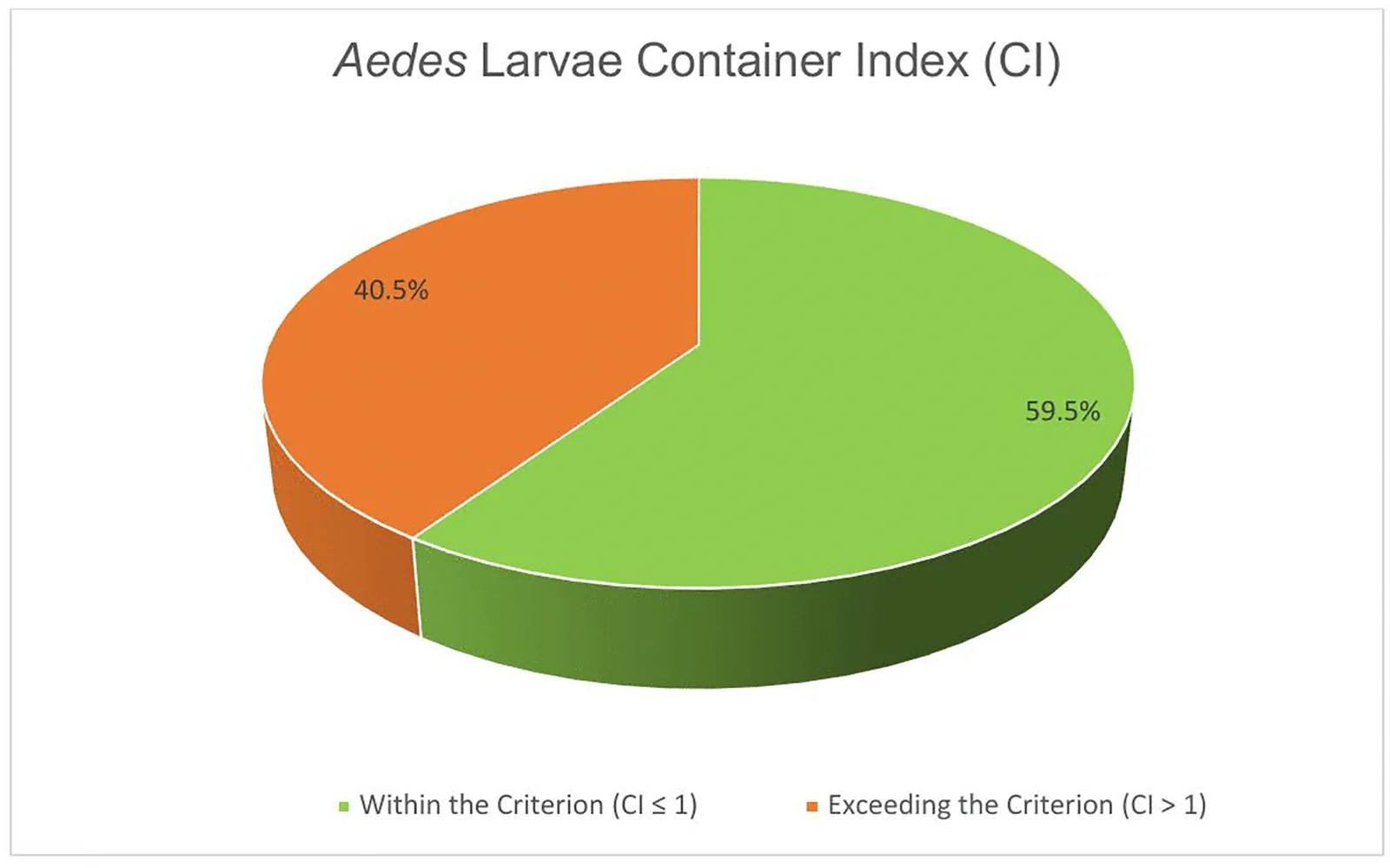
Percentage of the container index (CI) levels in water containers inspected for *Aedes* larvae among selected households in Sichon District, Nakhon Si Thammarat Province.

### 3.5 Environmental information

In terms of the environmental conditions surrounding the households of the respondents, 73.3% and 77.3% were single-house and one-story buildings, respectively. The majority, 53.6% of homes, were constructed with cement. A total of 643 households lived in clean and open areas that have no unused containers that could serve as mosquito breeding sites, and 593 households practiced waste segregation before disposal. Regarding water sources, the majority (68.6%) used tap water for household purposes, and 88.6% used purchased bottled drinking water. Additionally, 78.6% of the houses had indoor bathrooms. The environmental characteristics of the household representatives are presented in Table 4.

**Table 4:** Environmental characteristics of the household representatives who participated in the study (n =  824).

Environmental characteristics	Category	Number (%)
**House Distribution**	Detached house	604 (73.3)
	Clustered houses	220 (26.7)
**House Type**	Single-story/Two-story/Elevated house	820 (99.5)
	Commercial building/townhouse	4 (0.5)
**Construction Materials**	Cement and wood	382 (46.4)
	Cement	442 (53.6)
**Surrounding Area**	Area with garbage and discarded containers that serve as mosquito breeding sites	181 (22.0)
	Clean and open area, no discarded containers serving as mosquito breeding sites	643 (78.0)
**Waste Separation before Disposal**	Separated	593 (72.0)
	Not separated	231 (28.0)
**Water Sources for Household Use**	Tap water	565 (68.6)
	Natural water sources	259 (31.4)
**Drinking Water Sources**	Tap water and purchased bottled water	763 (92.6)
	Natural water sources	61 (7.4)
**Toilet Location**	Inside the house	648 (78.6)
	Outside the house	176 (21.4)

### 3.6 Understanding of dengue fever (UDS)

The highest score in understanding dengue solution was achieved by the question related to the identification of the disease, where 765 (92.8%) respondents provided the correct response. Only 155 (18.8%) respondents correctly answered the question about the risk of taking aspirin. Cumulatively, 586 (71.1%) had a low understanding of dengue, whereas 238 (28.9%) had a high understanding, as shown in Table 5.

**Table 5:** Understanding of dengue solutions among household representatives in Sichon District.

Questions	Response No. (%)	
	Correct	Incorrect
1. A high-grade fever lasting up to 7 days, petechiae, and a painful enlargement of the liver are signs of dengue infection	765 (92.8)	59 (7.2)
2. Causative agent of Dengue fever disease	586 (71.1)	238 (28.9)
3. If a person is protected from female *Aedes aegypti* mosquito bites, they will be safe from dengue	605 (73.4)	219 (26.6)
4. A person living in a high-risk area and infected with one dengue serotype can have lifelong immunity to that strain, though vulnerable to other serotypes, and could still be infected with other serotypes later, too	396 (48.1)	428 (51.9)
5. Having a high fever lasting for 2–7 days, nausea, vomiting, and abdominal pain shows the patient is in the fever stage	459 (55.7)	365 (44.3)
6. If a patient with dengue hemorrhagic fever presents with pain at the right lower costal margin, they are showing signs of hepatomegaly	387 (47.0)	437 (53.0)
7. If a patient with dengue hemorrhagic fever presents signs of shock from leakage of the plasma, they will have poor tissue perfusion, a weak pulse, and a narrowed pulse pressure	525 (63.7)	299 (36.3)
8. Dengue patients should avoid taking aspirin or non-steroidal anti-inflammatory drugs because they may cause gastritis and subsequent massive gastrointestinal or hepatic injury	155 (18.8)	669 (81.2)
9. If your neighbor presents with signs of poor tissue perfusion, a weak pulse, and clammy skin, you need to send them to the hospital	599 (72.7)	225 (27.3)
10. If your neighbor presents with a high-grade fever on day 1, you give one paracetamol every 6 h and a tepid sponge bath	539 (65.4)	285 (34.6)

### 3.7 The dengue larval index surveillance system (UDLSS)

Regarding responses to the UDLSS questions, less than half of the respondents answered nine questions correctly. 662 (80.3%) correctly responded to the question on eliminating mosquito breeding sites and diminishing larval indices to prevent massive occurrences of dengue fever, shown in Table 6. When computed for high and low understanding, only 10 (1.2%) of participants have a high understanding of the dengue larvae index surveillance system.

**Table 6:** Understanding of the dengue larvae index surveillance system among household representatives in Sichon District.

Questions	Response No. (%)	
	Correct	Incorrect
1. The number of larvae of female *Aedes aegypti* in the areas is determined by larval indices	410 (49.8)	414 (50.2)
2. Through the container index (CI), the value for identifying a dengue outbreak is calculated, and then the number of water containers infested with larvae is surveyed	241 (29.2)	583 (70.8)
3. Involvement of household representatives in the larval survey at the household level every week is a key	399 (48.4)	425 (51.6)
4. Insecticide Temephos (larvicide sand) may be used to eliminate larvae, but not to eliminate mosquito eggs	62 (7.5)	762 (92.5)
5. Use of lotion in the neighborhood works for the prevention of mosquito bites	420 (51)	404 (49)
6. Eliminating mosquito breeding sites and attempting to diminish the scores for the larval indices around the house works for the prevention of massive occurrences of dengue among people, especially in the village	662 (80.3)	162 (19.7)
7. Cleansing and scrubbing the edge of the container over the area that had water is effective in eliminating mosquitoes	214 (26)	610 (74)
8. Putting the water container upside down until the larvae can be seen, considering that the lifespan of a mosquito is 1–5 years	193 (23.4)	631 (76.6)
9. Red lime is used in the container if the water container capacity is 100 L	293 (35.6)	531 (64.4)
10. The larval indices surveillance system must be documented on the 25th day of every month in the “Violet book.”	148 (18)	676 (82)

### 3.8 Comparison of the UDS and UDLSS

Both the understanding of the dengue solution and the dengue Larval surveillance system were computed from their respective 10 questions. Accordingly, the prevalence of a high understanding of the dengue solution was 28.9%, whereas a high understanding of the dengue larval index surveillance system was only 1.2% among household representatives, as shown in Fig 2.

**Fig 2:**
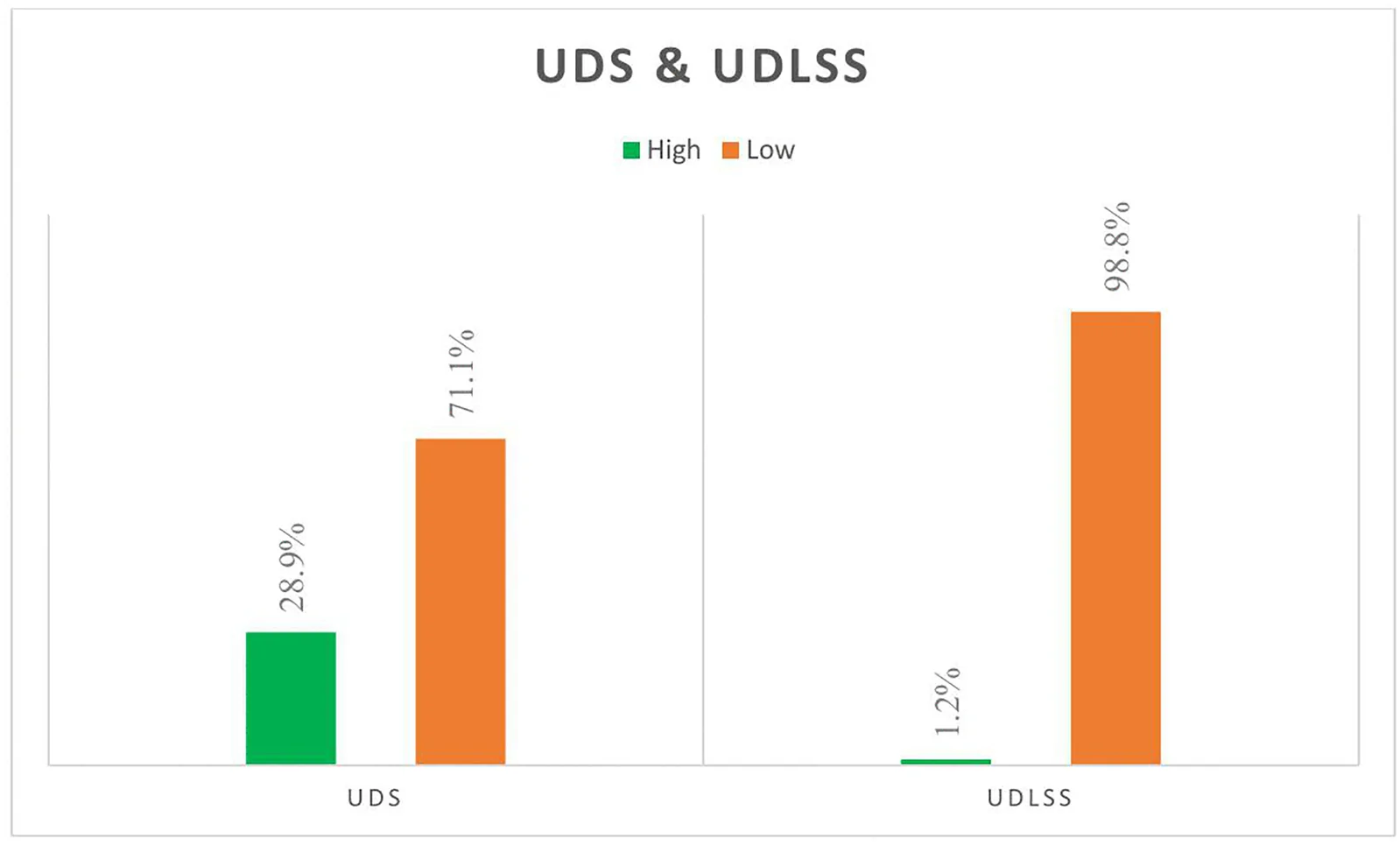
Understanding of dengue solutions and dengue larvae surveillance system among household representatives in Sichon District, Nakhon Si Thammarat Province.

### 3.9 Factors associated with UDS and UDLSS

Binary logistic regression was conducted after computing the understanding level of the dengue solution and the larval indices surveillance system. In the final model, attending training, the number of water containers, and the number of water containers positive for *Aedes* larvae were found to be associated with household representatives’ understanding of dengue solutions. The household representatives who attended dengue training were found to be two times (AOR 2.01, 95% CI [1.46, 2.78]) more likely to have a high understanding of dengue solutions than those who did not attend training. Household representatives with fewer water containers indoors and outside their home were found to be twice as likely (AOR 2.06, 95% CI [1.2, 3.54]) to have a higher understanding of dengue fever solutions than those with a greater number of water containers in and around their homes. Conversely, representatives from households with more water containers tested positive for *Aedes* larvae in their environment were 36% less likely (AOR 0.64, 95% CI [0.43, 0.97]) to possess a high understanding of dengue solutions than those from households without households with a low number of water containers with *Aedes* larvae. Factors associated with high UDLSS could not be examined using logistic regression because only ten participants met the predetermined criterion for high understanding. The results of the bivariate and multivariate binary logistic regression analyses are summarized in Table 7.

**Table 7:** Bivariate and multivariate analysis results for understanding of dengue solutions among household representatives in Sichon District, Nakhon Si Thammarat Province.

Characteristics	Category	Understanding of Dengue Solution		OR (95% confidence interval)	
		Low	High	COR (95%CI)	AOR (95%CI)
Age (years)	20–39	143	48	1	1
	40–59	334	141	1.26 [0.86, 1.84]	1.27 [0.84, 1.91]
	≥ 60	109	49	1.34 [0.84, 2.14]	1.58 [0.94, 2.65]
Education	Primary school and lower	272	102	1	1
	Secondary schools	176	72	1.09 [0.76, 1.56]	1.25 [0.86, 1.83]
	Vocational/Associate degree	46	18	1.04 [0.58, 1.88]	1.17 [0.64, 2.16]
	Bachelor’s and above	92	46	1.33 [0.88, 2.031]	1.58 [0.99, 2.51]
Training	Yes	174	110	2.04 [1.49, 2.78]	2.01 [1.46, 2.78] ^*^
	No	412	128	1	1
Larvae positive container	0	338	145	1	1
	1	57	29	1.19 [0.73, 1.93] [	1.0 [0.60, 1.66]
	2	37	19	1.20 [0.67, 2.15]	0.97 [0.52, 1.78]
	≥3	154	45	0.68 [0.46, 1.0]	0.64 [0.43, 0.97]^*^
Number of water containers	0-1	111	35	2.03 [1.20, 3.43]	2.06 [1.2, 3.54]*
	2-3	75	48	1.23 [0.81, 1.88] [1.1, 2.48]	1.49 [0.95, 2.33]
	≥3	400	155	1	1

* p < 0.05.

### 3.10 Qualitative results

The in-depth interview involved 13 household representatives; of these, ten were female and married, six were bachelor-qualified participants, and six of them attended dengue-related training. In the analysis, three themes emerged: recognition of dengue fever, preventive practices and risky seasons, and the importance of training.

#### 3.10.1 Theme 1. Recognition of dengue fever.

**Subtheme 1.1: Identification of symptoms of dengue fever**

Participants of the in-depth interview explained the signs, symptoms, and where to seek treatment when they or a family member or someone else develops the manifestation. The participants listed the signs as high fever, for three to five days, which is not lowered by sponging. The patient develops headaches, cannot eat or drink, experiences body aches, cannot move easily, and wants to rest all the time. When the fever begins to subside, the symptoms alleviate, and the patient resumes eating. The participant stated:

“There was only a high fever of 40 degrees Celsius; the patient could not eat at all when having a fever, but when the body temperature returned to normal, the patient started to eat again and regained appetite. The doctor treated according to the symptoms, gave only intravenous fluids, applied one patch, and prohibited food with black and red colors (FM4)” The thirteenth household representative raised, “Symptoms include vomiting, no bowel movement for two to three days, or in some cases, loose stools or diarrhea, loss of appetite, and red rashes or red bumps appearing on the body, and vomiting all day, about five to six times. Suddenly, red rashes appeared all over the body, completely covered” (FM13). First participant stated, “There was not a mild fever, it was a more severe kind of fever. I had pain under the left breast and could not lie on my left side. At first, I thought it was influenza.

The participants also mentioned the commonly used method of diagnosis and learned cautious practice after infection. They were tested for blood to confirm dengue fever, as well as to check for platelet count. The doctor tested continuously until he confirmed the absence of the virus from the blood, and the platelet count improved. As stated by the participant,

“Blood test showed that the virus was not yet complete, but the platelet count was low. They took blood for examination every six hours. The doctor treated according to the symptoms, gave only intravenous fluids, applied one patch, and prohibited food with black and red colors (FM4).” “Blood had to be drawn every day, FM1.” “There are some advantages for earlier infection. I feel like I have some immunity now, but if I do not repeat the same behavior, the chance of infection will be lower. I do not know how many times a person can get sick, but now I still feel scared every time I have a headache, always paranoid if it happens again (FM12).”

**Subtheme 1.2: Consequences of poor awareness**

The participants raised that the dengue virus infection needs immediate action. Sometimes people delay treatment due to poor awareness and mistakenly consider it as influenza. Therefore, it leads to complications like intense fever in the right lower chest and bumps on the body, and requires hospitalization with frequent blood tests. The participants stated that:

“There was pain under the left breast, making it impossible to lie on the left side. The patient did not know that these were symptoms of dengue fever FM12.” “There was no fever; it was a more severe kind of fever. Blood had to be drawn every day. I had pain under the left breast and could not lie on my left side. At first, I thought it was influenza. Sometimes we do not know what the symptoms of dengue fever are. If it is dengue fever, do not delay (FM1).” They feel their strength decreases, become anxious and fearful that other family members might get bitten by mosquitoes, become infected, and develop dengue fever as well as FM3.”

**Subtheme 1.3: Consequences of infection and delayed treatment-seeking**

Multiple impacts of dengue fever and delayed care-seeking for it were raised by the participants. When infected, the strength decreases, and the person becomes anxious and fearful that other family members might get bitten by mosquitoes, become infected, and develop dengue fever as well. Participants expressed these as:

“There are many impacts of dengue infection: financial expenses, work cannot be done, causing hardship, and also needing to take care of the children. If one of us is absent, it will be difficult (FM1).” “They also need to take extra care of their children who are ill. When the child becomes sick, they must stop attending school for about one to two weeks. The child had to stop going to school for two weeks during the exam period and failed in several subjects. Although the teacher was informed, after recovery, the child could not catch up with lessons, resulting in failing grades. The child felt sad, but the mother tried to help with the homework (FM3).”

#### 3.10.2 Theme 2. Preventive Practices and Risky Season.

The household representatives involved in the in-depth interview explained their understanding and experience regarding the dengue fever prevention practice and identifying risky seasons for high dengue transmission in their locality. The subthemes in this theme were preventing mosquito bites, removing stagnant water, and participation in a dengue prevention campaign.

**Subtheme 2.1: Preventing mosquito bites**

The household representatives emphasized the importance of protecting oneself and one’s family from mosquito bites. The prevention methods suggested by participants included using mosquito nets, spraying, and avoiding areas with high mosquito populations.

FM13 explained as “dengue, a disease transmitted by the female *Aedes* mosquito. This mosquito is highly dangerous when it bites, spreading the virus that causes dengue and can lead to outbreaks.”

The other participants expressed:

“Do not let mosquitoes bite at any time. Self-protection by sleeping under a mosquito net, being careful not to be bitten by mosquitoes, applying mosquito repellent, using mosquito spray, and avoiding areas where dengue patients are present and dark places where mosquitoes are abundant (FM2).” “Responding to VHV advice, officers and VHVs frequently go to the community and advise to spray mosquito repellent, and people in the village protect themselves (FM7).”

**Subtheme 2.2: Removing mosquito breeding sites**

The participants expressed their understanding of the prevention of dengue fever and the risky season, as the rainy season is a high-risk season, where water is accumulated in many containers and creating a good breeding site for mosquitoes. Removing those stagnant water sources is key to the prevention of mosquito breeding. Participants explained this as:

“Perform fogging to repel mosquitoes.”, “Got some Abate sand from the health center and put it in the jars in front of and beside the house (FM2, FM9).” "The outbreaks occur in breeding sites of *Aedes* mosquitoes and are influenced by weather conditions. The mosquito population is highest in the rainy season; it is less prevalent in the dry season because water accumulation is minimal then, unlike the rainy season when there is abundant stagnant water" (FM13). “To check around the house to see where mosquitoes are and eliminate breeding sources by pouring out standing water and turning containers upside down, people in the village can protect themselves as well as neighbors (FM5, FM7).”

#### 3.10.3 Theme 3: Importance of Training.

The training on dengue fever and the surveillance system is mostly given to village health volunteers, and then they train the households in the village. The purpose is to inform them about how to prevent *Aedes* mosquitoes, protect themselves and their families, and understand what type of mosquito causes dengue fever.

**Subtheme 3.1: Effects of training**

The household representatives participated in the in-depth interview explained the effect of training on personal protection and environmental management, to avoid mosquito breeding.

Participants expressed their experience regarding training as:

“Learned how to protect myself and my family correctly and understood what type of mosquito causes dengue fever (FM7).” “There are frequent dengue training sessions, which are good because we get to see and learn how others manage their homes and whether there are mosquito breeding sites (FM13).” “After attending the training, I learned how to eliminate mosquitoes by overturning water containers (FM8).” “I put the knowledge into practice by advising neighbors on eliminating mosquito breeding sites after training (FM7).”

**Subtheme 3.2: Need for training**

Participants also raised the need for training to know about dengue fever and the larval surveillance system. They expressed the perceived benefits of attending training sessions on dengue prevention and control, and the loss of important experience. The participants’ expressions of the need for training were:

“I think if I attend the training, it would be beneficial for myself and my child as well (FM4).” “If I attend, it will be beneficial because I can protect and take care of my family and nearby neighbors. I think attending the training would be very beneficial because there are many things I did not know, and sometimes the information on the internet is incorrect. Learning from experts is definitely better.” (FM2)

### 3.11 Integration of quantitative and qualitative findings

The qualitative and quantitative findings complement each other in the case of participants attending dengue training. Despite the quantitative results that revealed low UDS, very low UDLSS, poor water container management, and poor larvae index detection and measures, the household participants involved in the qualitative study expressed their understanding of dengue signs and symptoms, where to seek care, and the consequences of poor awareness and delayed treatment-seeking, as shown in Table 8.

**Table 8:** Joint display of quantitative and qualitative findings.

Quantitative findings	Qualitative themes	Integrated Interpretation (meta-inference)
238 (28.9%) of household representatives had a high understanding of dengue solutions	Recognition of dengue symptoms, causes, consequences, and appropriate preventive actions	The qualitative parts suggest that household representatives had experiential and practical knowledge of dengue, particularly regarding symptoms, transmission, and prevention. However, this knowledge does not necessarily translate into comprehensive understanding across the domains measured quantitatively. The richer qualitative descriptions may also partly reflect the purposive selection of participants with prior experience.
Only 10 (1.2%) had a high understanding of the Aedes larval surveillance system	Participants described preventing mosquito bites, removing stagnant water, identifying breeding sites, and recognizing the rainy season as a high-risk season	The apparent divergence reflects different dimensions of knowledge: participants demonstrated practical knowledge of source reduction and mosquito prevention but limited technical literacy regarding the formal larval surveillance system. Thus, the ability to identify and eliminate breeding sites should not be interpreted as an understanding of larval indices, surveillance procedures, or the interpretation of surveillance results.
Dengue-related training was associated with higher understanding of dengue solutions (AOR = 2.01, 95% CI 1.46–2.78)	A strong emphasis on the effects and perceived need for training, participants reported learning about mosquito identification, prevention, environmental management, and breeding-site elimination through training	The quantitative association and qualitative accounts converge to suggest that training is an important mechanism for strengthening understanding of dengue. However, the qualitative findings indicate that training may primarily enhance practical preventive knowledge. Therefore, suture training should incorporate technical larval surveillance concepts in addition to general prevention strategies.
Household representatives with fewer indoor or outdoor water containers were twice as likely to have high understanding of dengue solutions {AOR 2.06, 95% CI 1.20–3.54)	Participants recognized stagnant water and household water containers as important mosquito breeding sites and described practices to eliminate or manage it	The findings converge to suggest that greater understanding of dengue solutions is associated with greater awareness and management of household water containers. The qualitative part provides a behavioral explanation for the qualitative association, demonstrating how recognition of breeding sites may motivate source-reductions practices. However, the continued presence of water containers and larvae positive containers indicate that understanding alone may ot ensur complete environmental control or that other household or contextual factors may influence implementation.
High larval indices were observed across subdistricts and high numbers of *Aedes* larvae-positive containers were associated with lower understanding of dengue solutions	Participants recognized the rainy season as a period of increased mosquito abundance and described greater availability of stagnant water during this period.	the two strands of data converge in identifying environmental conditions, particularly water accumulation during the rainy season, as an important condition for larval growth. However, the recognition of seasonal risk does not appear to be sufficient to prevent high larval indices, suggesting a gap between risk awareness and sustained effective reduction practices of the sources.

## 4 Discussion

This mixed-methods study aimed to: 1) To describe the household representatives’ understanding of dengue solutions and the *Aedes* mosquito larvae index surveillance system; 2) To identify factors associated with household representatives’ understanding of dengue solutions and the *Aedes* mosquito larvae index surveillance system; 3) To explore the household representatives’ understanding of dengue solutions and the *Aedes* mosquito larvae index surveillance system.

In the current study, only 238 (28.9%) of the household representatives demonstrated a high understanding of dengue solutions, while an extremely small proportion, 10 (1.2%), showed adequate understanding of the larval surveillance system. Attending training related to dengue fever in the last 12 months, the number of water containers and *Aedes* larvae-positive containers were variables found to be associated with the household head’s understanding of dengue solutions in the multivariate analysis. None of the variables were found to be associated with the understanding of the *Aedes* larvae indices surveillance system.

A high level of understanding of dengue fever solutions was found only among 28.9% of household representatives in the study area. The current finding is lower than the findings of the study conducted on the dengue hotspot in Malaysia, where the good understanding of dengue fever was 50.7% [24] and the findings of the study conducted on the knowledge and practice of dengue fever among university students in Dhaka City, Bangladesh [25]. This finding is higher than a study conducted as the baseline of household heads’ understanding of dengue solutions, conducted before the implementation of the training program in high- and low-risk dengue villages in South Thailand, which was 6.3% [23] and on the primary school students’ knowledge (12.3%) [26]. But the finding is lower than the high school students’ understanding of dengue solutions (49.5%) in southern Thailand [20]. This variation may be attributed to differences in the population characteristics, the measurement tools, participants’ prior exposure to information sources, or dengue risk levels across areas.

The participants in the in-depth interview explained their understanding of dengue fever, which supports the above findings. Participants explained the common manifestations of dengue fever with recommended actions, and noted that weather conditions contribute to diseases like dengue fever, which is transmitted by the bite of infected female *Aedes* mosquitoes. They emphasized that these mosquitoes breed in stagnant water and their population surges during the rainy season when such breeding sites become abundant. As noted by one informant: “Dengue fever originates from *Aedes* mosquitoes, especially females. The disease spreads when mosquito breeding sites increase. Weather influences this; during the rainy season, mosquitoes are abundant, but during the dry season, there are fewer because rainfall creates more stagnant water, and …vomited all day about five to six times. Suddenly red rashes appeared all over the body, completely covered”, FM13. These complementary insights highlight that formal knowledge remains limited, and contextual understanding and disease experience contribute significantly to the findings among study participants.

The proportion of household representatives with a high understanding of the *Aedes* mosquito larval surveillance system was only 1.2%. This finding is lower than the baseline household representatives’ understanding of the larval indices surveillance system, conducted for the implementation of the training program in South Thailand, which was 4.4% [23], and a quasi-experimental study conducted in Pathum Thani province [15]. However, the current finding is higher than the high school students’ understanding of the larvae indices surveillance system, which was 0.2% [20].

The qualitative findings contrast with the quantitative findings, where participants of the qualitative findings described their experience as they actively engaged in campaigns, eliminated stagnant water, and used preventive measures. Participants stressed the engagement as, “we take care of our own houses, observe where there is stagnant water, and destroy it, and observe where there is stagnant water and remove it. The best way is to sleep under a mosquito net, FM7.” This might be because most households believe that *Aedes* mosquito larvae surveys are the responsibility of public health volunteers and the health care system, as stated by a participant: “VHVs spread information so that everyone knows, and VHVs regularly take care of larvae elimination, FM 5, FM6.” However, in reality, the household head is the most important person in surveying each household every 7 days for potential water sources that favor mosquito breeding. This indicates the potential gap in communication among the health system, village health volunteers, and the community. These findings also indicate poor translation of knowledge into dengue fever prevention practice among household representatives.

Household representatives who participated in dengue-related training within the past 12 months demonstrated a significantly better level of understanding of dengue fever solutions than those who did not attend training, with trained participants being twice as likely to have a high understanding. This finding is consistent with previous studies conducted among household heads, village health volunteers, and high school students in South Thailand, which reported a significant improvement in dengue-related understanding following educational intervention [13,23]. The qualitative findings further support this association, as participants emphasized the important role of training provided by health centers and village health volunteers in enhancing their knowledge of dengue prevention, water container management, and vector control. Participants underscored that, “I learned how to protect myself and my family correctly and understood what type of mosquito causes dengue fever, FM7”, while other noted, “There are frequent dengue training sessions, which are good because we get to see and learn how others manage their homes and whether there are mosquito breeding sites, FM 13.” These findings underscore the positive impact of training and emphasize the importance of such programs in enhancing understanding and skills, which in turn lead to timely measures for dengue fever and proactive preventive actions.

The number of water containers indoors and outdoors was found to be a significant determinant of household representatives’ understanding of dengue fever solutions. Participants who had fewer indoor and outdoor water containers in their household were twice as likely to have a high understanding of dengue fever. The studies conducted in Southeast Asia and Colombia also link household water management and container profiles to entomological risk and to the uptake of control measures, suggesting that households with many or large-purpose containers are less able to implement routine cleaning or covering, even when they know the prevention messages [24,27]. However, another earlier study found no direct association between overall dengue knowledge and the number of water containers [28], highlighting methodological and contextual differences, including season and knowledge domain, the current study focused on understanding what is beyond merely knowledge.

This finding is supported by the observation results of qualitative research participants’ living environment, and it can be further explained by the fact that those household representatives with a high understanding can manage or remove indoor and outdoor water containers to avoid acquiring dengue fever. In addition, the qualitative study finding supports this; participants explained their experience of removing water containers to prevent mosquito breeding as, “we check around the house to see where mosquitoes are there and eliminate breeding sources by pouring out standing water and turning containers upside down, people in the village can protect themselves (FM7).” This highlights that understanding the dengue solution enables the community in the prevention and control of dengue fever through control of *Aedes* mosquito breeding sites.

Regarding the association between the participants’ understanding of dengue solutions and the number of water containers positive for *Aedes* larvae, those participants whose household surroundings had two or more water containers that tested positive for *Aedes* larvae were 35% less likely to have a high understanding of dengue fever solutions. This finding goes in line with the KAP study conducted in Vietnam, and an entomological study in Jamaica showed larvae-positive container clusters in households with poor container management and weak source management practices associated with lower knowledge or constrained ability to act on knowledge [28,29]. The findings suggest that understanding dengue solutions is an important basis to act by eliminating potential mosquito breeding sites, even though understanding is not the sole predictor of the presence of higher *Aedes* larvae-positive containers.

The present study found that 40.5% of households had a container index (CI) exceeding the standard threshold, indicating that a considerable proportion of households had *Aedes* breeding conditions above the acceptable limits. Similar findings have been reported in various parts of Thailand and Southeast Asia, where household-level container index values often rise above the operational threshold during the rainy seasons or in areas with inadequate water management practices [23,30]. The container index of the current study is higher than the findings of a study conducted in Dire Dawa, Ethiopia, where the container index was 21% [31]. The observed pattern in Sichon aligns with previous evidence that container management practices and climatic variability are primary determinants of larval proliferation in endemic regions. Similar studies recommended focusing on integrated source reduction through improved water storage practices timed to precede peak transmission periods because CI greater than one is a potential early warning threshold for dengue transmission [30,32,33].

An important meta-inference from the mixed methods findings is that the community members may possess considerable experiential knowledge of dengue prevention without having corresponding technical literacy regarding the larval index surveillance system. The quantitative assessment showed that only 1.2% of household representatives met the predefined threshold for high understanding of larval surveillance, whereas qualitative participants described detailed experience of identifying stagnant water, eliminating breeding sites, and participating in dengue-prevention campaigns.

These findings are not necessarily contradictory because the two components captured different dimensions of knowledge. Moreover, the qualitative sample was purposively selected to provide rich information regarding their experience with dengue prevention activities. Thus, the integrated findings suggest that community experience with households’ vector-control practices may be substantially greater than understanding of formal surveillance concepts. This distinction is important because effective community-based dengue control requires both practical capacities to eliminate breeding sites and sufficient understanding of surveillance information to support meaningful participation in monitoring activities.

### 4.1 Strengths and limitations of the study

This study involved a mixed-methods approach, which is believed to generate rich information on the understanding of dengue fever solutions and the understanding of the *Aedes* larval index surveillance system. A mixed-methods approach also helped compare the quantitative and qualitative findings. In addition, the study employed environmental observations for the potential risk of mosquito breeding. This study also computed risk indicators of dengue fever transmission.

The cross-sectional nature of the study affects causal effect determination, and asking for experience of 12months might introduce minimal recall bias. In addition, the very low prevalence of high understanding of the Aedes larval index surveillance system was observed, with only 10 of 824 participants (1.2%). This resulted in too few outcome events to support reliable logistic regression analysis. Consequently, factors associated with high UDLSS couldn’t be meaningfully examined. The UDLSS findings should therefore be interpreted as descriptive rather than as evidence regarding determinants of larval surveillance understanding. The findings should also be interpreted in light of the higher proportion of women who participated in both the qualitative and quantitative components of the study.

## 5 Conclusions and recommendations

The level of understanding of household representatives of dengue solutions was low, and the *Aedes* larvae surveillance system was very low. The Container Index (CI), the Breteau Index (BI), and the House Index (HI) were above the threshold in all subdistricts in Sichon District, so they are at risk of dengue fever outbreaks. The number of water containers in and around the home, the number of *Aedes* larvae-positive containers, and training on dengue solutions and prevention were independent determinants of understanding of dengue solutions in the current study. Despite the participants in the qualitative study explaining their good understanding and practice, there was a critical gap in the understanding of dengue fever solutions and the *Aedes* larval surveillance system among household representatives.

Therefore, enhancing the network of village health volunteers and household representatives is essential to enable household representatives to actively participate in dengue fever prevention and control. Household representatives should play a key role in managing dengue fever and the environment with village health volunteers and district health offices. In addition to being aware of what to do for people suspected of having dengue fever, trained village health volunteers should supervise larval breeding sites while assisting the household representatives in water storage management, both indoors and outdoors, which could serve as *Aedes* mosquitoes breeding sites. Strengthening health education and awareness programs is essential to improve household representatives’ knowledge, motivation, and commitment to implementing the dengue prevention and control measures in their homes and communities. Public health authorities should also consider developing pictorial educational materials that enable community members to distinguish *Aedes* mosquitoes from other mosquito species and identify common breeding sites. The socioeconomic interpretation of water storage among residents should be considered when designing water container management strategies. More importantly, developing the workforce for vector-borne disease prevention and control would be an important strategy to address the community, in addition to village health volunteers.

## Supporting information

S1 Data Raw data SPSS file. (SAV)

S2 Data Raw data excel file. (XLSX)

S1 File Additional file. (DOCX)

## Abbreviations

BI: Brutaei Index
CI: Container Index
COVID: Corona Viral Disease
DenV: Dengue Virus
FM: Family Member
HI: Household Index
SPSS: Statistical Package for Social Sciences
UDLSS: Understanding of Dengue Larval Index Surveillance System
UDS: Understanding of Dengue Solutions
VHV: Village Health Volunteers
WHO: World Health Organization
