# Supplementary material for: Household representatives’ understanding of dengue solutions and larval indices surveillance systems in southern Thailand: A mixed methods study
Source: PLoS One. 2026 Oct 8;21(10):e0348142. doi: 10.1371/journal.pone.0348142 (PMC13648972; doi:10.1371/journal.pone.0348142)
Supplement: Supplementary file 1 [file pone.0348142.s003.docx]

| Subdistrict | Leading Health Service facilities | Total number of households | Number of households randomly selected |
| --- | --- | --- | --- |
|  |  |  |  |
| Sichon Subdistrict | Sichon Hospital | 2812 | 83 |
| Prang Subdistrict | Ban Chom Phibun Health Promoting Hospital | 4102 | 122 |
| Chalong Subdistrict | Ban Tha Kwai Subdistrict Health Promoting Hospital | 1056 | 31 |
|  | Ban Nai Don Subdistrict Health Promoting Hospital | 1373 | 41 |
| Sao Phao Subdistrict | Ton Riang Subdistrict Health Promoting Hospital | 3246 | 96 |
|  | Ban Na Lae Subdistrict Health Promoting Hospital | 1498 | 44 |
| Changed Subdistrict | Ban Plian Subdistrict Health Promoting Hospital | 1098 | 33 |
|  | Ban Tha Hin Subdistrict Health Promoting Hospital | 1307 | 39 |
| Si Khit Subdistrict | Ban Si Khit Subdistrict Health Promoting Hospital | 2323 | 69 |
|  | Ban Kret Raet Subdistrict Health Promoting Hospital | 909 | 27 |
| Theparat Subdistrict | Theparat Health Promoting Hospital | 1065 | 32 |
|  | Ban Nam Cha Health Promoting Hospital | 1789 | 53 |
| Khao Noi Subdistrict | Ban Khao Yai Health Promoting Hospital | 1776 | 53 |
| Thung Sai Subdistrict | Ban Khao Fai Health Promoting Hospital | 3413 | 101 |
| Total | | 27767 | 824 |

Strata used for proportional allocation and selection of study participants from Sichon District, Nakhon Si Thammarat

**Larval Indices in the Nine Subdistricts of Sichon District**

All nine subdistricts in Sichon District were identified as being at risk of dengue fever outbreaks. Analysis of the larval indices revealed that the breteau index (BI) ranged from 88 to 332 (standard BI ≤ 50), the house index (HI) ranged from 27 to 62 (standard HI ≤ 10), and the container index (CI) ranged from 13 to 30 (standard CI ≤ 1). Among all subdistricts, Thung Prang recorded the highest larval indices compared with the other subdistricts, as shown in the Table below.

**Table**: Larval Index Levels Compared with *Aedes* Mosquito Density per 1 Square Kilometer

| Subdistricts | Larval Indices | | | Number of Female *Aedes* mosquitoes per km^2^ |
| --- | --- | --- | --- | --- |
|  | BI | HI | CI |  |
| Sichon Subdistrict | 265.71 | 47.14 | 21.33 | 600,000 – 900,000 |
| Thung Prang Subdistrict | 332.38 | 61.90 | 30.82 | 700,000 – 900,000 |
| Chalong Subdistrict | 252.38 | 37.51 | 20.31 | 500,000 – 900,000 |
| Sao Phao Subdistrict | 209.32 | 56.78 | 27.16 | 600,000 – 900,000 |
| Plian Subdistrict | 233.90 | 52.54 | 18.01 | 500,000 – 900,000 |
| Si Khit Subdistrict | 626.92 | 50.56 | 28.41 | 700,000 – 900,000 |
| Thep Rat Subdistrict | 88.00 | 26.67 | 13.73 | 400,000 – 700,000 |
| Khao Noi Subdistrict | 186.00 | 48.00 | 25.45 | 600,000 – 800,000 |
| Thung Sai Subdistrict | 121.59 | 36.36 | 14.70 | 400,000 – 800,000 |
| Overall Sichon District | 217 | 46 | 21 | 600,000 – 900,000 |
