# Supplementary material for: Household representatives’ understanding of dengue solutions and larval indices surveillance systems in southern Thailand: A mixed methods study
Source: PLoS One. 2026 Oct 8;21(10):e0348142. doi: 10.1371/journal.pone.0348142 (PMC13648972; doi:10.1371/journal.pone.0348142)
Supplement: Supplementary file 2 [file pone.0348142.s005.docx]

**Response to the editor’s and reviewers’ comments**

**-**The ethics statement is removed from other sections except the methods section of the manuscript.

-We revised the data availability statement in the manuscript.

-The “financial disclosure” and “funding information” were updated to make them similar

-We amended the authorship list in the manuscript

-We included supporting information files caption at the end of the manuscript

-We checked and corrected the manuscript to meet the PLOS ONE style template, like headings, figures…

**Reviewer 1**-We italicized scientific nomenclatures consistently (*Aedes)* throughout the manuscript

**Reviewer 1**-We clarified how household representatives were selected and provided additional details regarding the stratified random sampling procedures employed in the study.

**Reviewer 1**-Incorporated the “development of simple educational materials” comment in the discussion section.

**Reviewer 1 & 2**-Revised the introduction section to remove redundancy and minimize the contents.

**Reviewer 2**-We revised and explained based on the author’s comment in lines 112 and 113

**Reviewer 2**-clarified the dengue solution meaning by alternative words in line 117

**Reviewer 2**-Reviewer’s comment regarding container index and larval index in lines 194 and 211, as well as in table 6, are more technical terms, so how can household representatives understand them? In the case of South Thailand, the subdistrict promotion hospitals train Village health volunteers. Then, village health volunteers train community members, teach the community regarding environmental management, and hold campaigns to remove water containers, which are potential breeding sites for *Aedes* mosquitoes. The village health volunteers also teach the community about the signs of dengue fever and what to do when family members have the signs of dengue fever, which we call the dengue solution in this study. Household representatives involved in the campaign learn the container index and the larvae index to become aware of these terms. But the actual number of water containers and the presence of larvae were observed and measured by the data collectors of this study.

**Reviewer 2:-** regarding the reviewer’s comment on the 10^th^ item of Table 6 in the result section: we appreciate the reviewer’s insightful comment. The item referring to the documentation of larval surveillance findings in the Violet Book was included because the Violet Book is a locally implemented community-based dengue surveillance tool used by village health volunteers in the study area, south Thailand. The intention was to assess familiarity with local dengue surveillance practices. Following the reviewer’s suggestions, we have revised the manuscript to clarify the purpose of this item and have acknowledged this issue as a limitation in the discussion.

**Reviewer 2:-Training and participant selection-related comments:** I thank the reviewer for this critical observation. We would like to clarify that participation in dengue-related training was neither an inclusion criterion nor used in participant selection. The question asks about attending training at least once in the last 12 months, which means having ever attended dengue fever-related training within the past 12 months, according to the authors’ explanation. Household representatives were selected through stratified random sampling from all subdistricts of Sichon District, regardless of their previous training experience. Therefore, the study population represents the community household representatives rather than a purposefully selected, trained group. The dengue-related training sessions were organized by the local public health authorities, including subdistrict health-promoting hospitals and the village health volunteer network, as part of routine dengue prevention and control activities implemented in the district.

**Reviewer 2:**-We put actual numbers before the proportion in the discussion section. The numbers and proportions were the findings of the quantitative study only.

**Reviewer 2:**- Revisions and paraphrasing were made in the recommendation and limitation sections to address both reviewers' comments.

**Reviewer 2:**- Responses for comments on Table 8 contents. We appreciate the reviewer’s comment and agree that reporting the percentage for a qualitative study is misleading. However, the 1.2% proportion was derived from a quantitative survey of 824 household representatives, in which only 10 participants correctly answered at least 8 of the 10 items. We have put the number of participants before the percentage to clarify the number of participants responded 80% and above with their respective proportions.

**Reviewer 2:**- The comment on line 544 of the discussion section: The authors did not mean that the general level of understanding was high. But those who had a high-level understanding of dengue fever solutions were 28.9%. We paraphrased again to avoid confusion in meaning.

**Reviewers 1 & 2:**- Regarding the use of the student population for comparison in the discussion section: We appreciate the reviewer’s insightful comment. We agree that direct comparisons between household representatives and student populations should be interpreted with caution because these groups differ in many characteristics. Due to the limited number of studies assessing the understanding of dengue solutions and larval surveillance systems, studies involving school students were referenced. These studies were conducted in Southern Thailand, a region with a similar dengue burden, and the intervention programs utilized schools as strategic settings for community-based dengue prevention and control efforts. However, we revised to make the primary comparison with other studies conducted among adults population and dengue spot areas.

**Reviewer 2:**- Discussion part, line 569 proportion-related comments. The authors appreciate the reviewer's comment. The proportion (1.2%) mentioned in the specified line was not the proportion of the qualitative study. It is the proportion from the quantitative part where only 10 participants responded as having a high level of UDLSS. We didn’t calculate the proportion for qualitative study participants.

**Reviewer 2:**- Comments on possible contradiction between results written in manuscript, for instance, in line 649 of discussion, “While quantitative results indicate low levels of understanding, particularly regarding larval surveillance systems”), Line 365 in the result section states that “662 (80.3%) correctly responded to the question on eliminating mosquito breeding sites and diminishing larval indices to prevent massive occurrences of dengue fever”. We thank the reviewer for the deep insight throughout the manuscript. There is no contradiction between these items. The data in the result section 80.3% was to show the specific response to question 6 of the larval surveillance system, which was the highest among 10 questions, whereas the interpretation in the discussion came from the cumulative responses on the understanding of the larval surveillance system.

**Reviewer 2:**- **minimizing the discussion by structuring in to findings**. It was a little bit challenging to minimize beyond the current one because of the nature of the mixed-method study, but we tried to minimize paragraphs by synthesizing the interpretation of a finding into a single paragraph.
