# Supplementary material for: Household representatives’ understanding of dengue solutions and larval indices surveillance systems in southern Thailand: A mixed methods study
Source: PLoS One. 2026 Oct 8;21(10):e0348142. doi: 10.1371/journal.pone.0348142 (PMC13648972; doi:10.1371/journal.pone.0348142)
Supplement: Supplementary file 3 [file pone.0348142.s006.docx]

**Response to the reviewer’s comments**

We sincerely thank the reviewers for their constructive and insightful comments, which have helped us improve the methodological rigor, clarity, and overall quality of the manuscript. We have carefully considered all comments and revised the manuscript accordingly, with each response provided point-by-point below and corresponding changes incorporated in the revised manuscript.

-The “financial disclosure” and “funding information” were updated to be the same in both the manuscript and the submission system. It was submitted to the editor with a cover letter submission of revised manuscript.

-We appreciate the reviewer's critical view of the typing and reference category error in the cross-tabulation of larvae-positive containers and UDS in Table 7. We corrected a typing error in Table 7. We checked the bivariate and multivariate analysis results again by rerunning them. The error was a typographical error. In addition, the reference group used in the bivariate analysis results for the larvae-positive container was the last category, as written in the table. This was wrongly written in the table; the first category is the reference group in both bivariate and multivariate analyses of the larvae-positive containers and the last category for the number of water containers. We have shown only multivariate results because most journals and reviewers request that it be removed from the manuscript, as it makes the manuscript or the table bulky and unattractive. If needed, we can add bivariate analysis results.

-Comments regarding mixed-methods integration: We appreciate the reviewer’s important and insightful comment. We agree that the integration of the qualitative and quantitative findings in the previous version was predominantly descriptive and that the integrated interpretation column did not sufficiently articulate higher-order meta-inferences. We therefore substantially revised the mixed -methods integration to move beyond simple description of findings and to examine convergence, complementarity, and divergence between the two strands of evidence.

-We appreciate the reviewer's critical view of the role of village health volunteers (VHV) in the sampling process. The main role of village health volunteers was simply showing the direction to the randomly selected household, not selecting the household. It was wrongly written that households were selected by village health volunteers. We accept the problem in the explanation and corrected it in the revised manuscript.

-Thank you for this important comment. We agree that the original description of the sample size calculation did not clearly distinguish between the sample size required for the overall implementation of the project and the analytical objectives of the present baseline mixed-methods study. The sample size of 824 household representatives was determined during the planning of the broader implementation project that followed the baseline mixed-methods assessment and was therefore not calculated specifically to power the two logistic regression models presented in the sample. We have revised the sample size determination section of the manuscript to state that the sample size was determined for the overall implementation project rather than specifically for cross-sectional regression analysis.

-We highly appreciate the reviewer’s comment regarding local operational tools, such as the "Violet book," red lime use, temephos, container cleaning details, and documentation on the 25th day of each month; in the case of South Thailand, the subdistrict promotion hospitals train Village health volunteers. Then, village health volunteers train community members, teach the community about environmental management, and hold campaigns to remove water containers, which are potential breeding sites for *Aedes* mosquitoes. The village health volunteers also teach the community about the signs of dengue fever and what to do when family members have the signs of dengue fever, which we call the dengue solution in this study. The item referring to the documentation of larval surveillance findings in the Violet Book was included because the Violet Book is a locally implemented community-based dengue surveillance tool used by village health volunteers in the study area, southern Thailand. The intention was to assess familiarity with local dengue surveillance practices. We clarified the purpose of this item and have acknowledged this issue as a limitation in the discussion.

-Thank you for this valuable comment. We agree that the term “dengue solution” is not a universally standardized public health term and that its use in the title and abstract could potentially be unclear to readers who are unfamiliar with the term. We appreciate the reviewer’s suggestion to use a more conventional term such as “dengue prevention and control.” However, dengue solution is the established terminology used in the broader project from which this study was derived and has also been used consistently in previous dengue-related studies conducted in southern Thailand. In the context of this project, “dengue solution” refers to the recommended care and preventive measures for dengue, including recognition of dengue symptoms and warning signs, appropriate care-seeking, source reduction, personal protection, and household- and community-level dengue prevention and control activities. We have therefore retained the term where it refers specifically to the project’s established construct to maintain consistency with the intervention, measurement instrument, and previous research. To improve clarity for readers, we put “care and preventive measures" in the abstract after the phrase dengue solutions in brackets.

-Thank you for this important methodological comment. We agree with the reviewer that the very low prevalence of high understanding of the Aedes larval index surveillance system (10/824, 1.2%) does not provide a sufficient number of outcome events to support a meaningful multivariable logistic regression analysis. We therefore agree that the absence of variables in the multivariable model should not be interpreted as evidence that no factors are associated with UDLSS. In response to this comment, we have revised the manuscript to clarify that multivariable logistic regression was not conducted for the UDLSS outcome because only 10 participants met the predefined criterion of high understanding.

-We are thankful for this important comment. We agree that an 80% cut-off may appear stringent, particularly for the UDLSS items, which we include locally relevant and technical aspects of the surveillance system. However, the ≥ 80% criterion was pre-defined based on Bloom's taxonomy of understanding, whereby a high proportion of correctly answered items was used to indicate a satisfactory/high level of understanding rather than merely recognition of individual facts. This criterion was also considered appropriate within our study context because dengue is endemic in the study area and household representatives have been exposed to ongoing community awareness and health education activities related to dengue prevention and control. Therefore, a high level of understanding was expected to reflect comprehensive knowledge sufficient for households to recognize and appropriately apply key dengue prevention and surveillance concepts. The ≥ 80% cut-off was defined a priori and applied consistently to both UDS and UDLSS; we retained it to preserve the conceptual and methodological consistency of the study and to avoid post hoc classification.

-Thank you for this methodological comment. We agree that the KR-20 is more appropriate than Cronbach’s alpha for evaluating internal consistency because UDS and UDLSS items were scored dichotomously as correct/incorrect, and reliability should be reported separately for the two measures. The instruments used in this study had previously undergone language adaptation and pretesting in a population comparable to the study population, using the same language and administration format as those used in the present study. The pretest was conducted before the main data collection to assess clarity, comprehensiveness, and appropriateness of the items for the target population. Accordingly, we re-evaluated the internal consistency of the 10-item UDS and 10-item UDLSS using KR-20, separately for pre-test data sets. In the pretest sample (n=30), the UDS demonstrated good internal consistency (KR-20=0.855), while the UDLSS demonstrated moderate internal consistency (KR-20= 0.65). However, the items affecting reliability in the UDLSS were checked and revised accordingly before the main study.

**-**Regarding the use of the student population for comparison in the discussion section: We appreciate the reviewer’s insightful comment. We agree that direct comparisons between household representatives and student populations should be interpreted with caution because these groups differ in many characteristics. Due to the limited number of studies assessing the understanding of dengue solutions and larval surveillance systems, studies involving school students were referenced. These studies were conducted in Southern Thailand, a region with a similar dengue burden, and the intervention programs used schools as strategic settings for community-based dengue prevention and control. However, we revised the manuscript to make the primary comparison with other studies conducted among adult populations and dengue spot areas.
