# Supplementary material for: Household representatives’ understanding of dengue solutions and larval indices surveillance systems in southern Thailand: A mixed methods study
Source: PLoS One. 2026 Oct 8;21(10):e0348142. doi: 10.1371/journal.pone.0348142 (PMC13648972; doi:10.1371/journal.pone.0348142)
Supplement: Peer review history

# Peer review: Decision Letter 0

**DOI:** 10.1371/journal.pone.0348142.r001

**Authors:** Vivekanandhan Perumal

8 Jun 2026

Dear Dr. Kinfe,

Thank you for submitting your manuscript to PLOS ONE. After careful consideration, we feel that it has merit but does not fully meet PLOS ONE’s publication criteria as it currently stands. Therefore, we invite you to submit a revised version of the manuscript that addresses the points raised during the review process.

Please submit your revised manuscript by Jul 23 2026 11:59PM. If you will need more time than this to complete your revisions, please reply to this message or contact the journal office at plosone@plos.org. When you're ready to submit your revision, log on to https://www.editorialmanager.com/pone/ and select the 'Submissions Needing Revision' folder to locate your manuscript file.

- A letter that responds to each point raised by the academic editor and reviewer(s). You should upload this letter as a separate file labeled 'Response to Reviewers'.
- A marked-up copy of your manuscript that highlights changes made to the original version. You should upload this as a separate file labeled 'Revised Manuscript with Track Changes'.
- An unmarked version of your revised paper without tracked changes. You should upload this as a separate file labeled 'Manuscript'.

If you would like to make changes to your financial disclosure, please include your updated statement in your cover letter. Guidelines for resubmitting your figure files are available below the reviewer comments at the end of this letter.

If applicable, we recommend that you deposit your laboratory protocols in protocols.io to enhance the reproducibility of your results. Protocols.io assigns your protocol its own identifier (DOI) so that it can be cited independently in the future. For instructions see: https://journals.plos.org/plosone/s/submission-guidelines#loc-laboratory-protocols. Additionally, PLOS ONE offers an option for publishing peer-reviewed Lab Protocol articles, which describe protocols hosted on protocols.io. Read more information on sharing protocols at https://plos.org/protocols?utm_medium=editorial-email&utm_source=authorletters&utm_campaign=protocols.

As the corresponding author, your ORCID iD is verified in the submission system and will appear in the published article. PLOS supports the use of ORCID, and we encourage all coauthors to register for an ORCID iD and use it as well. Please encourage your coauthors to verify their ORCID iD within the submission system before final acceptance, as unverified ORCID iDs will not appear in the published article. *Only*  the individual author can complete the verification step; PLOS staff *cannot*  verify ORCID iDs on behalf of authors.

We look forward to receiving your revised manuscript.

Kind regards,

Vivekanandhan Perumal, Ph.D

Academic Editor

PLOS One

Journal Requirements:

When submitting your revision, we need you to address these additional requirements.

1. Please ensure that your manuscript meets PLOS ONE's style requirements, including those for file naming. The PLOS ONE style templates can be found at

https://journals.plos.org/plosone/s/file?id=wjVg/PLOSOne_formatting_sample_main_body.pdf and https://journals.plos.org/plosone/s/file?id=ba62/PLOSOne_formatting_sample_title_authors_affiliations.pdf

2. Your ethics statement should only appear in the Methods section of your manuscript. If your ethics statement is written in any section besides the Methods, please delete it from any other section.

3. Please provide a complete Data Availability Statement in the submission form, ensuring you include all necessary access information or a reason for why you are unable to make your data freely accessible. If your research concerns only data provided within your submission, please write "All data are in the manuscript and/or supporting information files" as your Data Availability Statement.

4. We note that the grant information you provided in the ‘Funding Information’ and ‘Financial Disclosure’ sections do not match.

When you resubmit, please ensure that you provide the correct grant numbers for the awards you received for your study in the ‘Funding Information’ section.

5. Please amend your authorship list in your manuscript file to include author Aseb Arba Kinfe.

6. Please amend the manuscript submission data (via Edit Submission) to include author Aseb Arba.

7. Please include captions for your Supporting Information files at the end of your manuscript, and update any in-text citations to match accordingly. Please see our Supporting Information guidelines for more information: http://journals.plos.org/plosone/s/supporting-information.

8. Thank you for stating the following in the Acknowledgments Section of your manuscript:

The Authors express the deepest gratitude to Walailak University, Excellence Center for Public Health Research (EC-PHR). Our gratitude extends to VHVs and household representatives for their positive input on the success of the project.

We note that you have provided funding information that is not currently declared in your Funding Statement. However, funding information should not appear in the Acknowledgments section or other areas of your manuscript. We will only publish funding information present in the Funding Statement section of the online submission form.

Please remove any funding-related text from the manuscript and let us know how you would like to update your Funding Statement. Currently, your Funding Statement reads as follows:

The author(s) received no specific funding for this work.

Please include your amended statements within your cover letter; we will change the online submission form on your behalf.

9. If the reviewer comments include a recommendation to cite specific previously published works, please review and evaluate these publications to determine whether they are relevant and should be cited. There is no requirement to cite these works unless the editor has indicated otherwise.

Additional Editor Comments :

Dear Author,

After reviewing your manuscript, I can see that it has merit and potential. However, in its current form, the manuscript is not scientifically sound and contains several issues that need to be addressed. Therefore, I recommend that you carefully revise the manuscript and correct all identified errors based on the comments and suggestions provided by both reviewers.

[Note: HTML markup is below. Please do not edit.]

Reviewers' comments:

Reviewer's Responses to Questions

**Comments to the Author**

1. Is the manuscript technically sound, and do the data support the conclusions?

Reviewer #1: Yes

Reviewer #2: Partly

**********

2. Has the statistical analysis been performed appropriately and rigorously?

Reviewer #1: Yes

Reviewer #2: Yes

**********

3. Have the authors made all data underlying the findings in their manuscript fully available?

The PLOS Data policy

Reviewer #1: Yes

Reviewer #2: Yes

**********

4. Is the manuscript presented in an intelligible fashion and written in standard English?

Reviewer #1: Yes

Reviewer #2: Yes

**********

Reviewer #1: The manuscript entitled "Household Representatives’ Understanding of Dengue Solutions and Larval Indices Surveillance Systems in Southern Thailand" presents a comprehensive mixed-methods study that provides valuable insights into community knowledge gaps and factors influencing dengue prevention practices. The study combines robust quantitative data from a large household sample (n = 824) with qualitative interviews that enrich the interpretation of the findings. However, several issues require clarification and minor revisions before the manuscript can be considered for publication. Therefore, I recommend major revision. The authors are encouraged to address the following comments:

1. In introduction section, please provide a clearer overview of the dengue situation in the study region, including relevant epidemiological data such as recent dengue incidence and case numbers. In addition, explicitly state that Sichon District is a dengue-endemic area to better justify the rationale and significance of the study.

2. The manuscript would benefit from a more detailed description of the sampling methodology. Please clarify how household representatives were selected and provide additional details regarding the stratified random sampling procedures employed in the study.

3. The discussion section should include a more systematic comparison of the study findings with those reported in similar studies conducted in other dengue-endemic regions.

4. Given the study’s focus on household representatives’ understanding of dengue prevention and larval surveillance, the authors may consider recommending the development of simple educational materials, such as pictorial guides, to help community members distinguish Aedes mosquitoes from other mosquito species. This may enhance community participation and improve the effectiveness of larval surveillance activities.

5. Please ensure consistency in scientific nomenclature throughout the manuscript. All occurrences of Aedes (e.g., Aedes mosquitoes, Aedes aegypti) should be italicized according to standard scientific formatting conventions.

Reviewer #2: The topic of the manuscript of a great relevance, as dengue is a public health threat while individuals do have a role in the control of its spread through mosquito control interventions.

My main concern deals with the criteria of judgment used to assess the community understanding and knowledge on dengue and its vector (i.e., mosquito control measures). I may have misunderstood but it seems that questions deal with technical concept and administrative tasks. It is therefore predictable that a rural community (farmers, for instance) do not know such concepts and tasks. However, measuring whether the community knows how to prevent larva sites to spread and other vector control measures would have been more informative and adapted to this concept.

There are a few comments that may improve the quality of the manuscript.

- The Introduction is particularly long and could be shorten to more essential messages: for instance, the evolution of burden estimate is described in two long paragraphs, first worldwide then specifically in Thailand. At a time where scientific literature increases significantly, essentialising the message in the context would encourage readers.

- In the Introduction line 112, authors claim that a few studies exist in the investigation of “the understanding of both dengue solutions and the technical aspects of the larval surveillance system among household representatives”. That is an overstatement: many studies have. Please rephrase and specify.

- In the Introduction, line 113, “investigated the understanding of both dengue solutions and the technical aspects of the larval surveillance system among household representatives”. I find “dengue solutions” vague. What do the author mean exactly?

- In the Introduction line 117, when describing the objectives, I am concerned whether authors are measuring understanding of dengue prevention practices or understanding of entomological surveillance indicators.

- This concern remains in the Methods section. For instance, in line 194, have the authors asked to participants the meaning of “the Container Index (CI)” or the “larval indices”. I do not understand how relevant these questions would be, especially in a rural area targeting individuals who are neither entomologists nor specialist in vector control. Alternatively, authors may have asked questions using plain language, accessible to a common individual. This should be clarified. Same line 211 and so on.

- Related to this, in the Results section, table 6: questions are asked about the definition or meaning of “Container index”, which is very technical. How not being able to answer such technical question indicates a poor understanding of vector control interventions?

- Also in Table 6, it is asked whether “The larval indices surveillance system must be documented on the 25th day of every month in the “Violet book.” Is this common knowledge and duty in Thailand? Requiring registering each intervention for each health-related issues (here on dengue, but why not extending this method to other illnesses or antibiotic use, etc.) is not realistic. Why should household representatives be expected to know the administrative details of the larval surveillance system? Differently said: expecting general rural household representatives to know surveillance administration procedures is not typical public health design and often signals a measurement validity problem in the study.

- In the Results section, one third of participants attended at least more than one training on dengue fever in the past 12 months: this means the study focused on a well-trained, and aware population, which is not representative of the general population. Furthermore, who organized this training? Did the author select participants who benefited from this training purposely? Was it just random? Did this training include knowledge that could have helped participants to answer the questions of the current study? If so, we are here evaluating an understanding of a previously trained population…This study becomes a sort of training evaluation.

- In the Results section, Table 8: with only 13 household representatives being interviewed in-depth, I’d recommend using absolute number rather than proportions (1 out 13, and not 7.7 %). On this matter, Table 8 indicates proportion I do not understand. How did they reach 1.2%, as the proportion who had a high understanding of larval surveillance? Also, this subtheme (i.e., larval surveillance) shows a good understanding of the risk factor for larval proliferation “To check around the house to see where mosquitoes are and eliminate breeding sources by pouring out standing water and turning containers upside down, people in the village can protect themselves as well as neighbors (FM5, FM7).”The quantitative results (1.2% only) is in contradiction with FM5, 7.

- In the Discussion, line 544, the result is in contradiction with the interpretation (“The understanding level of dengue fever solutions was high in 28.9% of household representatives.”). In the following line, authors make a comparison with a score among students. How is that relevant? Where was the school located ? Did the students also benefit from training like this population? Same in line 548, 551: why comparing with students?

- In the Discussion line 569, authors keep presenting proportions without providing numbers, while the sample size is probably low. Interpretation of proportion based on 13 or so participants is risky. Further, the Results section and sentences used in the subtheme larva surveillance are in contradiction with such low proportion (1.2% for larval surveillance).

- In the Discussion section, the understanding of the larval surveillance system is described as low (e.g., line 649 “While quantitative results indicate low levels of understanding, particularly regarding larval surveillance systems”): this is in contradiction with the results. For instance, line 365 states that “662 (80.3%) correctly responded to the question on eliminating mosquito breeding sites and diminishing larval indices to prevent massive occurrences of dengue fever”.

- Overall, I’d structure the Discussion by topic. For instance, the importance of the training is repeated in different paragraphs, and so is the larval surveillance. This lack of structure undermines the interpretation of the results.

- In the Limitation paragraph, several are missing. The main one being the use of technical surveillance concepts to judge community knowledge. Also, there is an overrepresentation of women, married, which may not be representative of the general population. Please elaborate the weaknesses more in detail.

- In the conclusion, I am not very comfortable with a recommendation stated by the authors: “The legal and moral grounds should be established to penalize those household representatives who hesitate to take in dengue prevention and control measures in his or her surroundings.” Education, awareness raising etc. should be key: not criminalization.

**********

what does this mean?). If published, this will include your full peer review and any attached files.

If you choose “no”, your identity will remain anonymous but your review may still be made public.

**Do you want your identity to be public for this peer review?**  For information about this choice, including consent withdrawal, please see our Privacy Policy

Reviewer #1: No

Reviewer #2: No

**********

[NOTE: If reviewer comments were submitted as an attachment file, they will be attached to this email and accessible via the submission site. Please log into your account, locate the manuscript record, and check for the action link "View Attachments". If this link does not appear, there are no attachment files.]

To ensure your figures meet our technical requirements, please review our figure guidelines: https://journals.plos.org/plosone/s/figures

You may also use PLOS’s free figure tool, NAAS, to help you prepare publication quality figures: https://journals.plos.org/plosone/s/figures#loc-tools-for-figure-preparation.

NAAS will assess whether your figures meet our technical requirements by comparing each figure against our figure specifications.

# Author comment: Author response to Decision Letter 1

**DOI:** 10.1371/journal.pone.0348142.r002

16 Jun 2026

We appreciate the time and critical view of our manuscript and attached the areas where revision made and comments addressed in the separate file as, "response to reviewers" file. the details of revision points are detailed below:

Reviewer 1-We italicized scientific nomenclatures consistently (Aedes) throughout the manuscript

Reviewer 1-We clarified how household representatives were selected and provided additional details regarding the stratified random sampling procedures employed in the study.

Reviewer 1-Incorporated the “development of simple educational materials” comment in the discussion section.

Reviewer 1 & 2-Revised the introduction section to remove redundancy and minimize the contents.

Reviewer 2-We revised and explained based on the author’s comment in lines 112 and 113

Reviewer 2-clarified the dengue solution meaning by alternative words in line 117

Reviewer 2-Reviewer’s comment regarding container index and larval index in lines 194 and 211, as well as in table 6, are more technical terms, so how can household representatives understand them? In the case of South Thailand, the subdistrict promotion hospitals train Village health volunteers. Then, village health volunteers train community members, teach the community regarding environmental management, and hold campaigns to remove water containers, which are potential breeding sites for Aedes mosquitoes. The village health volunteers also teach the community about the signs of dengue fever and what to do when family members have the signs of dengue fever, which we call the dengue solution in this study. Household representatives involved in the campaign learn the container index and the larvae index to become aware of these terms. But the actual number of water containers and the presence of larvae were observed and measured by the data collectors of this study.

Reviewer 2:- regarding the reviewer’s comment on the 10th item of Table 6 in the result section: we appreciate the reviewer’s insightful comment. The item referring to the documentation of larval surveillance findings in the Violet Book was included because the Violet Book is a locally implemented community-based dengue surveillance tool used by village health volunteers in the study area, south Thailand. The intention was to assess familiarity with local dengue surveillance practices. Following the reviewer’s suggestions, we have revised the manuscript to clarify the purpose of this item and have acknowledged this issue as a limitation in the discussion.

Reviewer 2:-Training and participant selection-related comments: I thank the reviewer for this critical observation. We would like to clarify that participation in dengue-related training was neither an inclusion criterion nor used in participant selection. The question asks about attending training at least once in the last 12 months, which means having ever attended dengue fever-related training within the past 12 months, according to the authors’ explanation. Household representatives were selected through stratified random sampling from all subdistricts of Sichon District, regardless of their previous training experience. Therefore, the study population represents the community household representatives rather than a purposefully selected, trained group. The dengue-related training sessions were organized by the local public health authorities, including subdistrict health-promoting hospitals and the village health volunteer network, as part of routine dengue prevention and control activities implemented in the district.

Reviewer 2:-We put actual numbers before the proportion in the discussion section. The numbers and proportions were the findings of the quantitative study only.

Reviewer 2:- Revisions and paraphrasing were made in the recommendation and limitation sections to address both reviewers' comments.

Reviewer 2:- Responses for comments on Table 8 contents. We appreciate the reviewer’s comment and agree that reporting the percentage for a qualitative study is misleading. However, the 1.2% proportion was derived from a quantitative survey of 824 household representatives, in which only 10 participants correctly answered at least 8 of the 10 items. We have put the number of participants before the percentage to clarify the number of participants responded 80% and above with their respective proportions.

Reviewer 2:- The comment on line 544 of the discussion section: The authors did not mean that the general level of understanding was high. But those who had a high-level understanding of dengue fever solutions were 28.9%. We paraphrased again to avoid confusion in meaning.

Reviewers 1 & 2:- Regarding the use of the student population for comparison in the discussion section: We appreciate the reviewer’s insightful comment. We agree that direct comparisons between household representatives and student populations should be interpreted with caution because these groups differ in many characteristics. Due to the limited number of studies assessing the understanding of dengue solutions and larval surveillance systems, studies involving school students were referenced. These studies were conducted in Southern Thailand, a region with a similar dengue burden, and the intervention programs utilized schools as strategic settings for community-based dengue prevention and control efforts. However, we revised to make the primary comparison with other studies conducted among adults population and dengue spot areas.

Reviewer 2:- Discussion part, line 569 proportion-related comments. The authors appreciate the reviewer's comment. The proportion (1.2%) mentioned in the specified line was not the proportion of the qualitative study. It is the proportion from the quantitative part where only 10 participants responded as having a high level of UDLSS. We didn’t calculate the proportion for qualitative study participants.

Reviewer 2:- Comments on possible contradiction between results written in manuscript, for instance, in line 649 of discussion, “While quantitative results indicate low levels of understanding, particularly regarding larval surveillance systems”), Line 365 in the result section states that “662 (80.3%) correctly responded to the question on eliminating mosquito breeding sites and diminishing larval indices to prevent massive occurrences of dengue fever”. We thank the reviewer for the deep insight throughout the manuscript. There is no contradiction between these items. The data in the result section 80.3% was to show the specific response to question 6 of the larval surveillance system, which was the highest among 10 questions, whereas the interpretation in the discussion came from the cumulative responses on the understanding of the larval surveillance system.

Reviewer 2:- minimizing the discussion by structuring in to findings. It was a little bit challenging to minimize beyond the current one because of the nature of the mixed-method study, but we tried to minimize paragraphs by synthesizing the interpretation of a finding into a single paragraph.

Attachment Submitted filename: Response to reviewers.docx

# Peer review: Decision Letter 1

**DOI:** 10.1371/journal.pone.0348142.r003

**Authors:** Vivekanandhan Perumal

26 Aug 2026

Dear Dr. Kinfe,

Thank you for submitting your manuscript to PLOS One. After careful consideration, we feel that it has merit but does not fully meet PLOS One’s publication criteria as it currently stands. Therefore, we invite you to submit a revised version of the manuscript that addresses the points raised during the review process.

Please submit your revised manuscript by Oct 10 2026 11:59PM. If you will need more time than this to complete your revisions, please reply to this message or contact the journal office at plosone@plos.org. When you're ready to submit your revision, log on to https://www.editorialmanager.com/pone/ and select the 'Submissions Needing Revision' folder to locate your manuscript file.

- A letter that responds to each point raised by the academic editor and reviewer(s). You should upload this letter as a separate file labeled 'Response to Reviewers'.
- A marked-up copy of your manuscript that highlights changes made to the original version. You should upload this as a separate file labeled 'Revised Manuscript with Track Changes'.
- An unmarked version of your revised paper without tracked changes. You should upload this as a separate file labeled 'Manuscript'.

If you would like to make changes to your financial disclosure, please include your updated statement in your cover letter. Guidelines for resubmitting your figure files are available below the reviewer comments at the end of this letter.

If applicable, we recommend that you deposit your laboratory protocols in protocols.io to enhance the reproducibility of your results. Protocols.io assigns your protocol its own identifier (DOI) so that it can be cited independently in the future. For instructions see: https://journals.plos.org/plosone/s/submission-guidelines#loc-laboratory-protocols. Additionally, PLOS One offers an option for publishing peer-reviewed Lab Protocol articles, which describe protocols hosted on protocols.io. Read more information on sharing protocols at https://plos.org/protocols?utm_medium=editorial-email&utm_source=authorletters&utm_campaign=protocols.

As the corresponding author, your ORCID iD is verified in the submission system and will appear in the published article. PLOS supports the use of ORCID, and we encourage all coauthors to register for an ORCID iD and use it as well. Please encourage your coauthors to verify their ORCID iD within the submission system before final acceptance, as unverified ORCID iDs will not appear in the published article. *Only*  the individual author can complete the verification step; PLOS staff *cannot*  verify ORCID iDs on behalf of authors.

We look forward to receiving your revised manuscript.

Kind regards,

Vivekanandhan Perumal, Ph.D

Academic Editor

PLOS One

**Journal Requirements:**

1. If the reviewer comments include a recommendation to cite specific previously published works, please review and evaluate these publications to determine whether they are relevant and should be cited. There is no requirement to cite these works unless the editor has indicated otherwise.

**Additional Editor Comments:**

Dear Author, Please carefully revise the manuscript and fix the errors based on the reviewer comments.

[Note: HTML markup is below. Please do not edit.]

Reviewers' comments:

Reviewer's Responses to Questions

**Comments to the Author**

Reviewer #1: (No Response)

Reviewer #2: (No Response)

Reviewer #3: All comments have been addressed

Reviewer #4: (No Response)

Reviewer #5: All comments have been addressed

**********

2. Is the manuscript technically sound, and do the data support the conclusions?

Reviewer #1: Yes

Reviewer #2: Yes

Reviewer #3: Yes

Reviewer #4: No

Reviewer #5: Yes

**********

3. Has the statistical analysis been performed appropriately and rigorously?

Reviewer #1: Yes

Reviewer #2: Yes

Reviewer #3: Yes

Reviewer #4: No

Reviewer #5: Yes

**********

4. Have the authors made all data underlying the findings in their manuscript fully available?

The PLOS Data policy

Reviewer #1: Yes

Reviewer #2: Yes

Reviewer #3: Yes

Reviewer #4: Yes

Reviewer #5: Yes

**********

5. Is the manuscript presented in an intelligible fashion and written in standard English?

Reviewer #1: Yes

Reviewer #2: Yes

Reviewer #3: Yes

Reviewer #4: Yes

Reviewer #5: Yes

**********

Reviewer #1: After comparing the original manuscript, the revised version, and the authors’ point-by-point responses, I consider that the manuscript has substantially improved. While some comments were not fully addressed in the manner originally suggested, the authors have made a reasonable effort to respond to the reviewers’ concerns and have adequately addressed the major issues raised during the review process. The revised version is now considerably more cautious in scope, interpretation, and wording. In view of these revisions, I consider the manuscript suitable for publication.

Reviewer #2: See the document sent by email on the 17th of July 2026 to Ronalyn RAMOS, including detailed comments to the reveiwer's answers.

Reviewer #3: You have done a good research and good presentation. There is no doubt regarding research ethics, publication ethics and dual publication. Good luck for your research.

Reviewer #4: I regret that I cannot recommend this manuscript for publication in its current form. While the research question is important and the authors have collected a substantial dataset, the combination of a possible data error in Table 7, an effectively unanalyzable primary outcome for UDLSS, selection bias in sampling, construct validity concerns in the UDLSS instrument, and inconsistencies in funding and ethics statements undermines confidence in the reported findings. These are not minor issues that can be resolved through editing alone; they require substantial reanalysis, instrument revision, and reinterpretation. I therefore recommend rejection of the manuscript in its current form.

The most serious issue is in Table 7. The row for "Larvae positive container" appears to contain exactly the same cell counts as the "Education" row. This strongly suggests a copy-paste or coding error. If the larvae-positive container data are incorrect, then the reported association for larvae-positive containers, including the abstract's statement about "fewer larvae-positive containers (AOR 0.65…)," is unreliable. This must be corrected or removed before any further interpretation is possible.

A second major problem concerns the UDLSS outcome. Only 10 out of 824 participants (1.2%) had high understanding of the larval indices surveillance system. This near-zero prevalence makes logistic regression unstable, and indeed the authors report that no variable could be entered into multivariate analysis for UDLSS. This is not simply a limitation; it means the study cannot meaningfully identify factors associated with UDLSS. The authors should either use a less stringent outcome definition, analyse the score as a continuous or ordinal variable, or reframe the UDLSS component as descriptive only. As currently presented, the paper risks misleading readers into thinking "no factors were associated" when the analysis was underpowered to detect any association.

Related to this, both UDS and UDLSS were scored as correct/incorrect and then dichotomised at ≥80% correct as "high understanding." This threshold is not well justified. For the UDLSS items, many are highly local or technical, so an 80% threshold may be unrealistically strict and may not reflect meaningful differences in understanding. A continuous score, median split, or latent class analysis would be more informative and would avoid the extreme imbalance seen with UDLSS.

The sample size calculation also needs clarification. The authors used G*Power with an effect size of 0.15, df of 2, and power of 0.95. This appears to be based on a linear multiple regression or similar model, not clearly on logistic regression with binary outcomes. The authors also state that an expected 7% improvement from 33.9% was used, but this is not coherently linked to the reported effect size. For two binary outcomes, especially one with only 1.2% prevalence, the sample size should have been justified using expected events per variable or simulation. The current justification does not support the analyses performed.

There is also a concern about sampling and selection bias. The manuscript states: "VH leaders then guided the data collectors in selecting household representatives in their respective villages according to the inclusion criteria." This means village health volunteers helped select participants, which can introduce selection bias, social desirability, and deviations from random sampling. The stratified random sampling procedure is described, but the role of VH leaders in actual household selection is not compatible with strict random selection. In addition, the qualitative participants were purposively selected because they had experience with dengue fever and larval index surveillance. This likely explains why qualitative themes suggest more awareness than quantitative data. This selection bias is not adequately acknowledged in the integration.

The construct validity of the UDLSS questionnaire is also questionable. Several items are highly specific to local operational tools, such as the "Violet book," red lime use, temephos, container cleaning details, and documentation on the 25th day of each month. These items measure familiarity with local surveillance procedures rather than a general understanding of larval indices or vector surveillance. A respondent could have a reasonable conceptual understanding of larval surveillance but still score poorly because they are not familiar with specific local tools. This threatens the validity of the UDLSS outcome and should be more explicitly addressed. The authors mention this partially in the limitations, but the instrument itself may need revision.

There are also several statistical and reporting issues. The association with "fewer larvae-positive containers" is reported as AOR 0.65 for category 2 versus ≥3. This is not the same as "fewer larvae-positive containers" overall and does not show a consistent dose–response pattern. The unadjusted OR for 0–1 water containers is 0.81, but the adjusted OR is 2.06. Such a large reversal should be explained and checked for instability or collinearity. No bivariate table is provided for UDLSS, and the statement that no variables were candidates for multivariate analysis may be misleading given only 10 high cases. Finally, the clustering by subdistrict or village is not accounted for in the logistic models, which may underestimate standard errors.

The mixed-methods integration is also less developed than it could be. Although the study uses a convergent parallel design and a joint display, the integration is mostly descriptive. Table 8 lists quantitative findings and qualitative themes side by side, but the "integrated interpretation" is often a general statement rather than a true meta-inference. For example, the apparent contradiction between low quantitative understanding and rich qualitative awareness is not adequately explained. The qualitative sample was deliberately enriched with experienced participants, which likely explains part of the divergence, but this is not discussed in the integration.

There is also a clear inconsistency in the financial disclosure. The submission form states: "The author(s) received no specific funding for this work." However, the manuscript states: "This research work is funded by the Excellence Center for Public Health Research, School of Public Health, Walailak University." This must be corrected.

The discussion still compares household representatives with primary school children, high school students, and university students. Even though the authors partially revised this, these comparisons remain. These groups differ in age, education, exposure, and responsibility for household vector control. Comparisons with adult household members or community-based samples would be more appropriate.

The phrase "dengue solution" is used throughout, but it is not a standard public health term. The authors define it as "care and preventive measures," but the title and abstract may confuse readers. A clearer term such as "dengue prevention and control" or "dengue knowledge and management" would improve clarity.

Finally, the authors report Cronbach's alpha of 0.70. However, Cronbach's alpha is not ideal for binary correct/incorrect items. A Kuder–Richardson formula 20 or test–retest reliability would be more appropriate. The manuscript also does not report reliability separately for the UDS and UDLSS subscales.

Reviewer #5: The study is very interesting and showed important points concerning the dengue transmitting mosquito that is widely distributed. Also the study revealed how natives understand such a situation. It was interesting to show believes of some people! Does a mosquito knows the difference? Any how, the work is good. The authors followed the reviewers' comments precisely.

**********

what does this mean?). If published, this will include your full peer review and any attached files.

If you choose “no”, your identity will remain anonymous but your review may still be made public.

**Do you want your identity to be public for this peer review?**  For information about this choice, including consent withdrawal, please see our Privacy Policy

Reviewer #1: No

Reviewer #2: No

Reviewer #3: **Yes:**  Nitikorn Phoosuwan

Reviewer #4: No

Reviewer #5: No

**********

[NOTE: If reviewer comments were submitted as an attachment file, they will be attached to this email and accessible via the submission site. Please log into your account, locate the manuscript record, and check for the action link "View Attachments". If this link does not appear, there are no attachment files.]

To ensure your figures meet our technical requirements, please review our figure guidelines: https://journals.plos.org/plosone/s/figures

You may also use PLOS’s free figure tool, NAAS, to help you prepare publication quality figures: https://journals.plos.org/plosone/s/figures#loc-tools-for-figure-preparation.

NAAS will assess whether your figures meet our technical requirements by comparing each figure against our figure specifications.

# Author comment: Author response to Decision Letter 2

**DOI:** 10.1371/journal.pone.0348142.r004

3 Sep 2026

Response to the reviewer’s comments

We sincerely thank the reviewers for their constructive and insightful comments, which have helped us improve the methodological rigor, clarity, and overall quality of the manuscript. We have carefully considered all comments and revised the manuscript accordingly, with each response provided point-by-point below and corresponding changes incorporated in the revised manuscript.

-The “financial disclosure” and “funding information” were updated to be the same in both the manuscript and the submission system. It was submitted to the editor with a cover letter submission of revised manuscript.

-We appreciate the reviewer's critical view of the typing and reference category error in the cross-tabulation of larvae-positive containers and UDS in Table 7. We corrected a typing error in Table 7. We checked the bivariate and multivariate analysis results again by rerunning them. The error was a typographical error. In addition, the reference group used in the bivariate analysis results for the larvae-positive container was the last category, as written in the table. This was wrongly written in the table; the first category is the reference group in both bivariate and multivariate analyses of the larvae-positive containers and the last category for the number of water containers. We have shown only multivariate results because most journals and reviewers request that it be removed from the manuscript, as it makes the manuscript or the table bulky and unattractive. If needed, we can add bivariate analysis results.

-Comments regarding mixed-methods integration: We appreciate the reviewer’s important and insightful comment. We agree that the integration of the qualitative and quantitative findings in the previous version was predominantly descriptive and that the integrated interpretation column did not sufficiently articulate higher-order meta-inferences. We therefore substantially revised the mixed -methods integration to move beyond simple description of findings and to examine convergence, complementarity, and divergence between the two strands of evidence.

-We appreciate the reviewer's critical view of the role of village health volunteers (VHV) in the sampling process. The main role of village health volunteers was simply showing the direction to the randomly selected household, not selecting the household. It was wrongly written that households were selected by village health volunteers. We accept the problem in the explanation and corrected it in the revised manuscript.

-Thank you for this important comment. We agree that the original description of the sample size calculation did not clearly distinguish between the sample size required for the overall implementation of the project and the analytical objectives of the present baseline mixed-methods study. The sample size of 824 household representatives was determined during the planning of the broader implementation project that followed the baseline mixed-methods assessment and was therefore not calculated specifically to power the two logistic regression models presented in the sample. We have revised the sample size determination section of the manuscript to state that the sample size was determined for the overall implementation project rather than specifically for cross-sectional regression analysis.

-We highly appreciate the reviewer’s comment regarding local operational tools, such as the "Violet book," red lime use, temephos, container cleaning details, and documentation on the 25th day of each month; in the case of South Thailand, the subdistrict promotion hospitals train Village health volunteers. Then, village health volunteers train community members, teach the community about environmental management, and hold campaigns to remove water containers, which are potential breeding sites for Aedes mosquitoes. The village health volunteers also teach the community about the signs of dengue fever and what to do when family members have the signs of dengue fever, which we call the dengue solution in this study. The item referring to the documentation of larval surveillance findings in the Violet Book was included because the Violet Book is a locally implemented community-based dengue surveillance tool used by village health volunteers in the study area, southern Thailand. The intention was to assess familiarity with local dengue surveillance practices. We clarified the purpose of this item and have acknowledged this issue as a limitation in the discussion.

-Thank you for this valuable comment. We agree that the term “dengue solution” is not a universally standardized public health term and that its use in the title and abstract could potentially be unclear to readers who are unfamiliar with the term. We appreciate the reviewer’s suggestion to use a more conventional term such as “dengue prevention and control.” However, dengue solution is the established terminology used in the broader project from which this study was derived and has also been used consistently in previous dengue-related studies conducted in southern Thailand. In the context of this project, “dengue solution” refers to the recommended care and preventive measures for dengue, including recognition of dengue symptoms and warning signs, appropriate care-seeking, source reduction, personal protection, and household- and community-level dengue prevention and control activities. We have therefore retained the term where it refers specifically to the project’s established construct to maintain consistency with the intervention, measurement instrument, and previous research. To improve clarity for readers, we put “care and preventive measures" in the abstract after the phrase dengue solutions in brackets.

-Thank you for this important methodological comment. We agree with the reviewer that the very low prevalence of high understanding of the Aedes larval index surveillance system (10/824, 1.2%) does not provide a sufficient number of outcome events to support a meaningful multivariable logistic regression analysis. We therefore agree that the absence of variables in the multivariable model should not be interpreted as evidence that no factors are associated with UDLSS. In response to this comment, we have revised the manuscript to clarify that multivariable logistic regression was not conducted for the UDLSS outcome because only 10 participants met the predefined criterion of high understanding.

-We are thankful for this important comment. We agree that an 80% cut-off may appear stringent, particularly for the UDLSS items, which we include locally relevant and technical aspects of the surveillance system. However, the ≥ 80% criterion was pre-defined based on Bloom's taxonomy of understanding, whereby a high proportion of correctly answered items was used to indicate a satisfactory/high level of understanding rather than merely recognition of individual facts. This criterion was also considered appropriate within our study context because dengue is endemic in the study area and household representatives have been exposed to ongoing community awareness and health education activities related to dengue prevention and control. Therefore, a high level of understanding was expected to reflect comprehensive knowledge sufficient for households to recognize and appropriately apply key dengue prevention and surveillance concepts. The ≥ 80% cut-off was defined a priori and applied consistently to both UDS and UDLSS; we retained it to preserve the conceptual and methodological consistency of the study and to avoid post hoc classification.

-Thank you for this methodological comment. We agree that the KR-20 is more appropriate than Cronbach’s alpha for evaluating internal consistency because UDS and UDLSS items were scored dichotomously as correct/incorrect, and reliability should be reported separately for the two measures. The instruments used in this study had previously undergone language adaptation and pretesting in a population comparable to the study population, using the same language and administration format as those used in the present study. The pretest was conducted before the main data collection to assess clarity, comprehensiveness, and appropriateness of the items for the target population. Accordingly, we re-evaluated the internal consistency of the 10-item UDS and 10-item UDLSS using KR-20, separately for pre-test data sets. In the pretest sample (n=30), the UDS demonstrated good internal consistency (KR-20=0.855), while the UDLSS demonstrated moderate internal consistency (KR-20= 0.65). However, the items affecting reliability in the UDLSS were checked and revised accordingly before the main study.

-Regarding the use of the student population for comparison in the discussion section: We appreciate the reviewer’s insightful comment. We agree that direct comparisons between household representatives and student populations should be interpreted with caution because these groups differ in many characteristics. Due to the limited number of studies assessing the understanding of dengue solutions and larval surveillance systems, studies involving school students were referenced. These studies were conducted in Southern Thailand, a region with a similar dengue burden, and the intervention programs used schools as strategic settings for community-based dengue prevention and control. However, we revised the manuscript to make the primary comparison with other studies conducted among adult populations and dengue spot areas.

Attachment Submitted filename: Response to reviewer 2.docx

# Peer review: Decision Letter 2

**DOI:** 10.1371/journal.pone.0348142.r005

**Authors:** Vivekanandhan Perumal

23 Sep 2026

Household Representatives’ Understanding of Dengue Solutions and Larval Indices Surveillance Systems in Southern Thailand: A Mixed Methods Study

PONE-D-26-17769R2

Dear Dr. Kinfe,

We’re pleased to inform you that your manuscript has been judged scientifically suitable for publication and will be formally accepted for publication once it meets all outstanding technical requirements.

Within one week, you’ll receive an e-mail detailing the required amendments. When these have been addressed, you’ll receive a formal acceptance letter and your manuscript will be scheduled for publication.

An invoice will be generated when your article is formally accepted. Please note, if your institution has a publishing partnership with PLOS and your article meets the relevant criteria, all or part of your publication costs will be covered. Please make sure your user information is up-to-date by logging into Editorial Manager at Editorial Manager® and clicking the ‘Update My Information' link at the top of the page. For questions related to billing, please contact billing support.

If your institution or institutions have a press office, please notify them about your upcoming paper to help maximize its impact. If they’ll be preparing press materials, please inform our press team as soon as possible -- no later than 48 hours after receiving the formal acceptance. Your manuscript will remain under strict press embargo until 2 pm Eastern Time on the date of publication. For more information, please contact onepress@plos.org.

Kind regards,

Vivekanandhan Perumal, Ph.D

Academic Editor

PLOS One

Additional Editor Comments (optional):

The manuscript has been carefully revised in response to the reviewers’ comments. The authors have adequately addressed the major concerns, and the manuscript is now clear, coherent, and scientifically sound. The revisions have improved the overall quality and presentation of the work. Therefore, I recommend acceptance for publication in its present form.

Reviewers' comments:

Reviewer's Responses to Questions

**Comments to the Author**

Reviewer #4: All comments have been addressed

**********

2. Is the manuscript technically sound, and do the data support the conclusions?

Reviewer #4: Yes

**********

3. Has the statistical analysis been performed appropriately and rigorously?

Reviewer #4: Yes

**********

4. Have the authors made all data underlying the findings in their manuscript fully available?

The PLOS Data policy

Reviewer #4: Yes

**********

5. Is the manuscript presented in an intelligible fashion and written in standard English?

Reviewer #4: Yes

**********

Reviewer #4: Thanks to the author for their efforts. All my comments resolved.

I recommend for acceptance.

**********

what does this mean?). If published, this will include your full peer review and any attached files.

If you choose “no”, your identity will remain anonymous but your review may still be made public.

**Do you want your identity to be public for this peer review?**  For information about this choice, including consent withdrawal, please see our Privacy Policy

Reviewer #4: No

**********

# Editor report: Acceptance letter

**DOI:** 10.1371/journal.pone.0348142.r006

**Authors:** Vivekanandhan Perumal

PONE-D-26-17769R2

PLOS One

Dear Dr. Kinfe,

I'm pleased to inform you that your manuscript has been deemed suitable for publication in PLOS One. Congratulations! Your manuscript is now being handed over to our production team.

At this stage, our production department will prepare your paper for publication. This includes ensuring the following:

* All references, tables, and figures are properly cited

* All relevant supporting information is included in the manuscript submission,

* There are no issues that prevent the paper from being properly typeset

You will receive further instructions from the production team, including instructions on how to review your proof when it is ready. Please keep in mind that we are working through a large volume of accepted articles, so please give us a few days to review your paper and let you know the next and final steps.

Lastly, if your institution or institutions have a press office, please let them know about your upcoming paper now to help maximize its impact. If they'll be preparing press materials, please inform our press team within the next 48 hours. Your manuscript will remain under strict press embargo until 2 pm Eastern Time on the date of publication. For more information, please contact onepress@plos.org.

You will receive an invoice from PLOS for your publication fee after your manuscript has reached the completed accept phase. If you receive an email requesting payment before acceptance or for any other service, this may be a phishing scheme. Learn how to identify phishing emails and protect your accounts at https://explore.plos.org/phishing.

If we can help with anything else, please email us at customercare@plos.org.

Thank you for submitting your work to PLOS One and supporting open access.

Kind regards,

PLOS One Editorial Office Staff

on behalf of

Dr. Vivekanandhan Perumal

Academic Editor

PLOS One
